# Supplementary material for: Optimizing quantum gates towards the scale of logical qubits
Source: Nat Commun. 2024 Mar 18;15:2442. doi: 10.1038/s41467-024-46623-y (PMC10948820; doi:10.1038/s41467-024-46623-y)
Supplement: Supplementary file 1 — Supplementary Information [file 41467_2024_46623_MOESM1_ESM.pdf]

Optimizing quantum gates towards the scale of logical qubits

Paul V. Klimov[1],* Andreas Bengtsson[1], Chris Quintana[1], Alexandre Bourassa[1], Sabrina Hong[1], Andrew Dunsworth[1], Kevin J. Satzinger[1], William P. Livingston[1], Volodymyr Sivak[1], Murphy Yuezhen Niu[1], Trond I. Andersen[1], Yaxing Zhang[1], Desmond Chik[1], Zijun Chen[1], Charles Neill[1], Catherine Erickson[1], Alejandro Grajales Dau[1], Anthony Megrant[1], Pedram Roushan[1], Alexander N. Korotkov[1,2], Julian Kelly[1], Vadim Smelyanskiy[1], Yu Chen[1], and Hartmut Neven[1]
[1]*Google AI, Mountain View, CA, USA and*
[2]*Department of Electrical and Computer Engineering, University of California, Riverside, CA, USA*

CONTENTS

Supplementary Notes 1

1. Control system 1

2. Characterization system 2

3. Algorithm error estimator 2
  3.1. Construction 2
  3.2. Error components 3
  3.3. Runtime 4
  3.4. Extensions to other hardware 4

4. Training the estimator 5
  4.1. Bounding model capacity 5
  4.2. Sampling training data 5
  4.3. Training 5
  4.4. Accuracy 6
  4.5. Innacuracy sources 6

5. Optimization system 8
  5.1. Optimization variables 8
  5.2. Optimization bounds 10
  5.3. Optimization parameters 10
    5.3.3. Seed strategy 10
    5.3.3. Traversal strategy 10
    5.3.3. Scope 11
    5.3.3. Inner loop optimizer 12
  5.4. Optimization runtime budget 12
  5.5. Optimization runtime scalability 13

6. Benchmarking system 13

7. Simulation environment 14
  7.1. Simulated processor generative model 15
  7.2. Simulated characterization data 16

8. Additional experimental details 16
  8.1. Experimental controls 16
  8.2. Impact of drift 16
  8.3. Healing experiment 16
  8.4. Metrology experiment 16
    8.4.4. Qualitative analysis 16
    8.4.4. Quantitative Analysis 19
  8.5. Scaling experiment 20
  8.6. Stitching experiment 20
  8.7. Algorithm specificity 20
  8.8. Dependencies 20

Supplementary References 26

1. CONTROL SYSTEM

Here we overview our control system to offer context for where our optimization system exists within our quantum computing stack (Supplementary Fig. 1). The following sections describe most of these components in more detail. Our control system learns the control-electronics signals needed to execute quantum gates for some target quantum algorithm (A). It can be split into the characterization, optimization, calibration, and benchmarking systems. The characterization system measures data (D) that are believed to be necessary to operate and estimate the performance of quantum gates over qubits' operable frequency ranges, including qubits' flux sensitivities versus frequency ($\sim T_\phi^{-1}$ spectra), energy-relaxation rates versus frequency (T_1^{-1} spectra), parasitic stray coupling parameters (χ coupling), and frequency-pulse distortion parameters (δ parameters). The optimization system then estimates the quantum algorithm's error (E) around that data and optimizes it over gate frequencies (F). The calibration system[1–3] then learns the control parameters necessary to execute gates at the optimized frequency configuration (F^*). Finally, the benchmarking system evaluates the performance of the configuration via one or more benchmarking algorithms. If calibration failures were not encountered and if benchmarks exceed some standard of high performance, the quantum algorithm is executed.

* corresponding author, pklimov@google.com

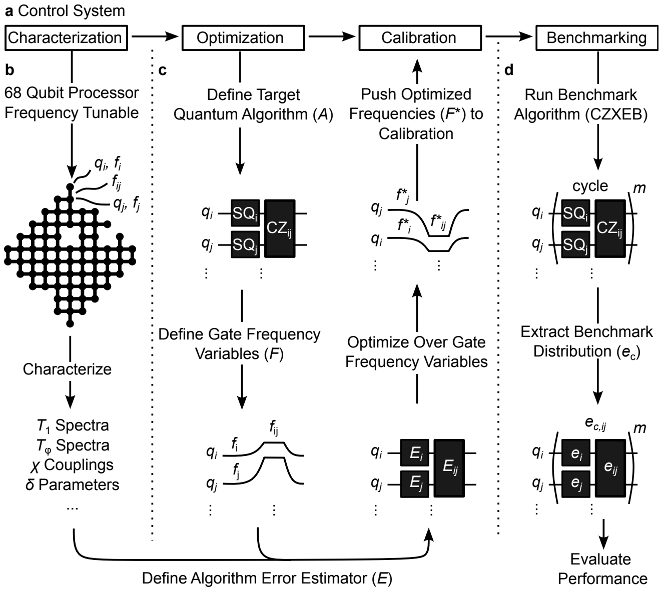

Supplementary Fig. 1. **Control system**. Overviews of our (a) control system and it's (b) characterization, (c) optimization, and (d) benchmarking components.

2. CHARACTERIZATION SYSTEM

The characterization system[1,2] interrogates quantum computational elements - including qubits, couplers, and readout resonators - and the control electronics. The output is the characterization data D, which includes:

- Gate trajectory parameters

  SQ gate length: $t_{\mathrm{SQ}} = 25$ ns.

  CZ gate length: $t_{\mathrm{CZ}} = 34$ ns.

  $\mathcal{F}$ State-dependent CZ frequency trajectory[4].

- Hardware and control parameters

  T_1 relaxation time versus frequency (i.e. spectra), from which we estimate relaxation errors.

  $\frac{df}{d\phi}$ flux sensitivity spectra, from which we estimate $\sim T_\phi$ dephasing errors.

  χ parasitic stray coupling parameters, from which we estimate stray coupling errors due to parasitic coupling between nearest- and next-nearest-neighbor qubits.

  δ frequency-pulse parameters, from which estimate pulse distortion errors due to large frequency excursions during CZ gates.

The characterization data are used to construct optimization hard bounds and the algorithm error estimator's error components, as discussed next.

3. ALGORITHM ERROR ESTIMATOR

The algorithm error estimator can be represented by a variety of models (e.g. a linear model, a neural network, or a quantum simulation). The only strict requirement for optimization is that the estimator be representative of the performance of the target quantum algorithm. For this study, we append several requirements - physicality, speed, and accuracy. These are competing interests that are difficult to satisfy simultaneously. In the following sections we describe how we construct an estimator that can satisfy them.

3.1. Construction

We define the algorithm error estimator $E(F|A, D)$ by making several simplifying assumptions:

1. A quantum algorithm's error can be decomposed into a sum over the gate error estimators $E_g(F_g|A, D)$ of its constituent gates $g \in A$ (i.e. CZXEB with 2 qubits and m cycles comprises $2m$ SQ and m CZ gate error estimators). F_g is the subset of F that are relevant to E_g. This assumption is motivated by the digital error model, which states that gate errors can be added when they are small and not correlated in space or time[5], and which was validated in the context of random circuits[1].

2. A gate's error can be decomposed into a sum over algorithm-independent error components $\epsilon_{g,m}(F_{g,m}|D)$, each of which corresponds to a distinct physical error mechanism $m \in M$. $F_{g,m}$ is the subset of F_g that are relevant to estimating $\epsilon_{g,m}$. This assumption should apply in the limit of small and uncorrelated error components. Error components should be defined for all known error mechanisms, including those that go beyond the assumptions of the digital error model (e.g. stray coupling errors, which are correlated in space). Furthermore, they should be defined for all possible algorithmic contexts (e.g. stray coupling components for all possible combinations of parasitically coupled gates executed concurrently).

3. The effect of implementing an arbitrary gate within an arbitrary quantum algorithm can be fully encompassed by algorithm-dependent weights $w_{g,m}(A)$. One unique weight is assigned to, and multiplies, each error component. The weights filter error components by algorithmic relevance (e.g. weights corresponding to stray coupling errors are only non-zero for concurrent gates) and relative contribution (e.g. weights corresponding to dephasing errors are lower in algorithms with dynamical decoupling than without).

4. The weights can be trained on benchmarks that are sufficiently representative of the target quantum algorithm. Empirical training injects algorithmic context and can compensate for some of the simplifying assumptions made above and inaccuracies in the error components.

These assumptions lead to the decomposition:

$$E(F|A,D) = \sum_{g\in A} E_g(F_g|A,D) = \sum_{g\in A}\sum_{m\in M} w_{g,m}(A)\epsilon_{g,m}(F_{g,m}|D) \tag{1}$$

This decomposition establishes a powerful and broadly applicable framework:

- Compatible with physicality, speed, and accuracy.
- Error components can be added as new error mechanisms are discovered.
- Error components can be interchanged to transfer the estimator between hardware architectures (Supplementary Note 3.4).
- Weights can be re-trained to transfer the estimator between quantum algorithms.
- Optimization variables (F here) can be interchanged to adapt the estimator to other control variables, provided that the error components are defined in terms of those variables.
- Equivalent to the basis-expansion method in machine learning[6–8], bridging the domains and facilitating knowledge transfer.

The assumption that errors can be added in this way is expected to break down in certain limits - particularly in highly structured quantum circuits. Fortunately, methods like randomized compiling[9–11] offer the potential to recompile those algorithms into compliance. Finally, even when our assumptions cannot be fulfilled, we expect that our algorithm error estimator can still serve as a valuable optimization proxy.

3.2. Error components

We define error components for the dephasing[12–14], relaxation[13,15–18], stray coupling[19], and pulse distortion[20,21] error mechanisms. We leverage textbook physics, published literature, and metrology of our quantum and classical control hardware. Furthermore, since runtime is a critical scaling consideration, we engineer error components for speed via software optimizations, physical simplifications, and mathematical approximations, some of which trade against accuracy.

Here we provide key error components $\epsilon_{g,m}$ for gates g and mechanisms m, the relevant frequencies $F_{g,m}$ and characterization data D, and how they scale with processor size N. We note that each error mechanism is segmented into multiple error components as described in detail below. When referencing a mechanism, we consider all such components together.

1. Single qubit gate error components $g = \mathrm{SQ}_i$
   (a) $m =$ Dephasing error on SQ_i due to q_i.
   $\epsilon_{g,m}(F_{g,m}) \propto t_{\mathrm{SQ}}/T_{\phi,i}(f_i) \propto t_{\mathrm{SQ}}\frac{df_i}{d\phi_i}$.
   $F_{g,m} = \{f_i\}$
   $D =$ Flux-sensitivity spectrum for q_i, $\frac{df_i}{d\phi_i}$ and SQ_i length, t_{SQ}.
   Scale: N (1 per SQ gate)
   (b) $m =$ Relaxation error on SQ_i due to q_i.
   $\epsilon_{g,m}(F_{g,m}) \propto t_{\mathrm{SQ}}/T_{1,i}(F_{g,m})$.
   $F_{g,m} = \{f_i\}$
   $D =$ Relaxation spectrum for q_i, $T_{1,i}(F_{g,m})$ and SQ_i length, t_{SQ}.
   Scale: N (1 per SQ gate)
   (c) $m =$ Stray coupling error on SQ_i due to SQ_j.
   $\epsilon_{g,m}(F_{g,m}) \propto \mathrm{Lorentzian}(F_{g,m};\chi_{ij})$.
   $F_{g,m} = \{f_i, f_j\}$
   $D =$ Stray coupling parameters between SQ_i and SQ_j, χ_{ij}.

$$\text{Scale: } 32N \begin{cases} N \text{ SQ gates} \\ 8 \text{ parasitic qubits each} \\ 4 \text{ collisions each} \end{cases} \tag{2}$$

2. Two qubit gate error components $g = \mathrm{CZ}_{ij}$.
   (a) $m =$ Dephasing error on CZ_{ij} due to q_i undergoing some state-dependent frequency trajectory $\mathcal{F}_i(F_{g,m})$.
   $\epsilon_{g,m}(F_{g,m}) \propto \int_{\mathcal{F}_i(F_{g,m})} \frac{df}{d\phi}\frac{df}{dt}^{-1} df$
   $F_{g,m} = \{f_i, f_j, f_{ij}\}$
   $D =$ Flux-sensitivity spectrum $\frac{df}{d\phi}$ for q_i and CZ_{ij} frequency trajectory $\mathcal{F}_i$.

$$\text{Scale: } \sim 8N \begin{cases} \sim 2N \text{ CZ gates} \\ 4 \text{ input states each} \end{cases} \tag{3}$$

   (b) $m =$ Relaxation error on CZ_{ij} due to q_i undergoing some state-dependent frequency trajectory $\mathcal{F}_i(F_{g,m})$.
   $\epsilon_{g,m}(F_{g,m}) \propto \int_{\mathcal{F}_i(F_{g,m})} T_{1,i}^{-1}(f)\frac{df}{dt}^{-1} df$
   $F_{g,m} = \{f_i, f_j, f_{ij}\}$
   $D =$ Relaxation spectrum $T_{1,i}(f_i)$ for q_i and CZ_{ij} frequency trajectory $\mathcal{F}_i$.

$$\text{Scale: } \sim 8N \begin{cases} \sim 2N \text{ CZ gates} \\ 4 \text{ input states each} \end{cases} \tag{4}$$

(c) $m =$ Stray coupling error on CZ_{ij} due to CZ_{kl}.
$\epsilon_{g,m}(F_{g,m}) \propto \mathrm{Lorentzian}(F_{g,m}; \chi_{ij,kl})$.
$F_{g,m} = \{f_i, f_j, f_k, f_l, f_{ij}, f_{kl}\}$
$D =$ Stray coupling parameters between CZ_{ij} and CZ_{kl}, $\chi_{ij,kl}$.

$$\text{Scale: } \sim 896N \begin{cases} \sim 2N \text{ CZ gates} \\ 14 \text{ parasitic qubits each} \\ \sim 32 \text{ collisions each} \end{cases} \tag{5}$$

(d) $m =$ Frequency-pulse distortion error on CZ_{ij} due to q_i's excursion from f_i to $\sim f_{ij}$.
$F_{g,m} = \{f_i, f_{ij}\}$
$D =$ Frequency-pulse distortion parameters for CZ_{ij}, δ_{ij}.
Scale: $\sim 2N$ (1 per CZ gate)

In total, an N qubit processor has $\sim 10^3 N$ error components. Our $N = 68$ processor has $\sim 4 \times 10^4$ error components. This number is large due to the high granularity with which we segment error components:

- Each error mechanism is generally segmented into single- and two-qubit error components (e.g. 1a and 2a above for dephasing).
- Two-qubit error components typically integrate errors over frequency trajectories $\mathcal{F}$ according to the hardware implementation and the local temporal order of gates within the quantum algorithm. For CZXEB, they may integrate over a trapezoidal trajectory that links idles and interactions.
- Each error component is segmented by gate. For example, we consider 68 (N) components for SQ dephasing (1a above) and 109 ($\sim 2N$) components for CZ pulse distortion (2d above).
- Some error components are segmented by input state. For example, we consider the 4 input states $|00\rangle, |01\rangle, |10\rangle, |11\rangle$ for CZ dephasing and relaxation (2a and 2b above).
- Stray coupling error components are segmented by the number of parasitically coupled qubits and the number of frequency collisions each (1c and 2c above). For example, we consider 8 parasitic qubits for each SQ gate (nearest and next-nearest neighbors) and 14 parasitic qubits for each CZ gate (nearest and next-nearest neighbors, excluding the CZ qubits). Furthermore, we consider 4 collisions between each pair of parasitically coupled SQ gates (all possible collisions between the qubits' respective $|0\rangle \leftrightarrow |1\rangle$ and $|1\rangle \leftrightarrow |2\rangle$ transition frequencies) and 32 collisions between each pair of parasitically coupled qubits executing CZ gates.

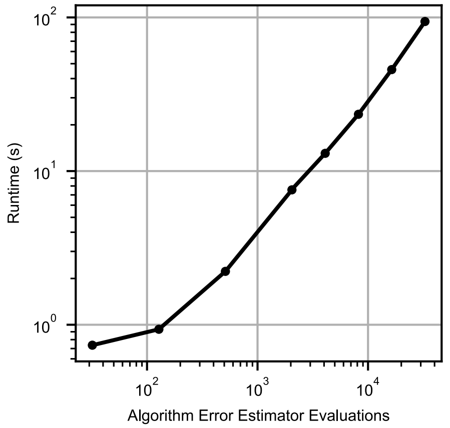

Supplementary Fig. 2. **Algorithm error estimator runtime**. Runtime versus the number evaluations of our algorithm error estimator on a high-performance desktop. The estimator is defined over 177 gate frequency variables and comprises $\sim 40,000$ error components. Despite its large scale, it can still be evaluated $> 100\times$/second.

3.3. Runtime

The majority of optimization runtime is spent evaluating error components. To estimate how quickly we can evaluate our error components, we consider evaluation runtimes for our $N = 68$ processor's 177 gate-frequency variable algorithm error estimator on a high-performance desktop (Supplementary Fig. 2). We can evaluate the estimator > 100/second, which translates into $\lesssim 1/$ second for evaluating all error components of a gate on average.

3.4. Extensions to other hardware

Extending the algorithm error estimator to control optimization problems in other hardware will require defining new error components. While the connection between error components in our transmon qubits[22] and other superconducting qubits[23] is reasonably clear, the connection to entirely different hardware is not. To illustrate the versatility of our framework, we connect our error components to control objectives when choreographing the spatial trajectories of reconfigurable atom arrays[24,25].

The objective of minimizing atom loss and heating by avoiding crossing spatial trajectories is analogous to our stray coupling components, which penalize crossing frequency trajectories. The objective of minimizing atom move distance is analogous to our pulse distortion components, which penalize large frequency excursions. The objective of minimizing atoms' vertical extent is similar to our dephasing components, which squeeze frequencies towards their maxima. Similar connections exist to control problems in other hardware, for example shuttling electrons in quantum dots[26–29] or shuttling ions in ion traps[30–33].

4. TRAINING THE ESTIMATOR

When training the estimator, we must ensure that it generalizes to unseen configurations of our processor. In turn, we take significant care in mitigating the risk of overfitting[6–8,34]. In traditional statistics and machine learning applications, overfitting is often combated by reducing the complexity of a model by discarding or constraining correlated features - e.g. via principal component analysis - or by suppressing them during training - e.g. via regularization. These approaches are most compatible with models that don't necessarily require interpretability, such as neural networks. However, they are incompatible with our requirement that the estimator be physical. Namely, we must keep all physically relevant error components, even though some of them may be correlated in some scenarios. Next we formalize the overfitting problem and then develop training-data sampling and model-training protocols to overcome it.

4.1. Bounding model capacity

Model capacity is an important concept in machine learning that measures the expressivity of a model and is often associated with the number of trainable parameters[34]. It is desirable to select a model that has a high enough capacity to be able to represent complex patterns but low enough capacity to reduce the risk of overfitting and the amount of training data necessary.

For the algorithm error estimator, we equate the model capacity with the number of independent trainable weights. Within this definition, the capacity of our $N = 68$ algorithm error estimator is $\sim 4 \times 10^4$, since each error component generally has an independent trainable weight. Furthermore, given local engineered and parasitic coupling, we expect the capacity to scale linearly with processor size.

Given the considerations mentioned above, taking all weights to be independent trainable parameters is neither practical nor scalable. To bound the capacity of our estimator, we make several reasonable assumptions:

- Homogeneity: The parameters of our processor are homogeneous enough such that weights for the same error components for the same gate types are equal. For example 1(a) above, this assumption leads to $w_{g,m} = w_{g',m}$ for $g =\mathrm{SQ}_i$ and $g' =\mathrm{SQ}_j$.

- Symmetry: The weights of some error components must be equal due to symmetry. For example 2(a) above, this assumption leads to $w_{g,m} = w_{g,m'}$, where m and m' correspond to the respective trajectories $\mathcal{F}_i$ for the input states $|0,1\rangle$ and $|1,0\rangle$.

By applying these assumptions, we reduce our algorithm error estimator's capacity from $\sim 4 \times 10^4$ to 16. Furthermore, capacity does not grow with processor size. We thus believe this is a scalable strategy for bounding the capacity of our algorithm error estimator.

4.2. Sampling training data

Building a sufficiently large and diverse training dataset such that the trained estimator generalizes to complex and unseen scenarios is a complex problem in benchmarking and statistics[6,8]. Towards that end, we leverage the flexibility of our quantum processor architecture to accomplish the following:

- Multiple training targets: We train on both SQRB and CZXEB benchmarks.

- Isolate error components: We employ the frequency-tunability of our architecture to benchmark gates in configurations of variable complexity. For example, to isolate the relaxation and dephasing error components, we benchmark gates in sparse configurations with negligible stray coupling. As another example, to controllably introduce stray coupling, we benchmark gates in denser configurations with only nearest- or next-nearest neighbor gates benchmarked simultaneously.

- Boost statistical leverage: We employ the frequency-tunability of our architecture to vary the relative strength of each error component. For example, we benchmark gates near and far from their respective qubits' maximum frequencies to generate data with high leverage in dephasing. As another example, we benchmark gates near and far from two-level-system (TLS) defects to generate data with high leverage in relaxation.

For this study, we sampled ~ 6500 benchmarks, averaging > 100 training benchmarks per independent trainable weight, over several weeks. Although time consuming, the trained estimator remains viable over months, and is trivial to refine as benchmarks accumulate over time. Furthermore, the runtime cost of acquiring training data in parallel scales inversely with processor size (i.e. since the number of gates that can be benchmarked in parallel scales as $\sim N$), which is especially favorable from a scalability perspective. Finally, we believe that if a sufficiently complete understanding of a processor and the control system is available, the training data may be augmented via generative modeling methods similar to those used to generate our simulated processors[35].

4.3. Training

To train the algorithm error estimator's weights, we developed an iterative supervised learning protocol[36]. It progressively trains and constrains distinct subsets of the estimator's weights over multiple training iterations

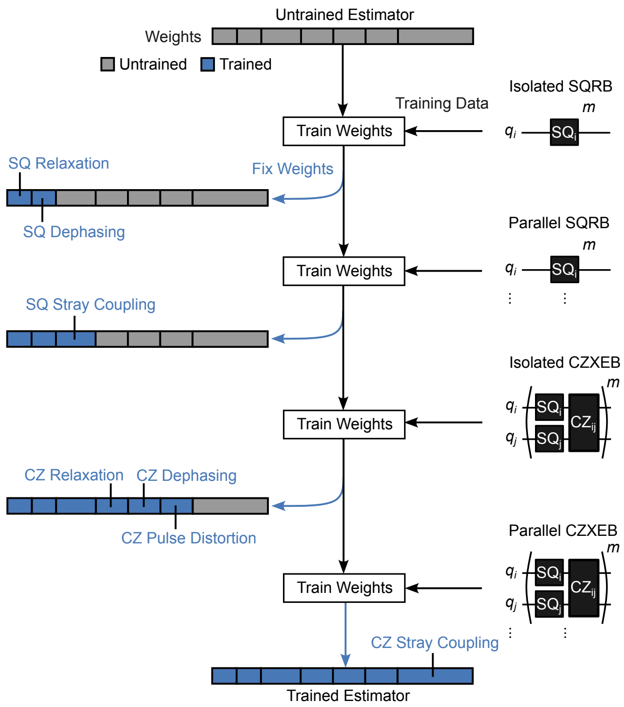

Supplementary Fig. 3. **Iterative training protocol**. We train the algorithm error estimator over multiple iterations on benchmarks of increasing complexity. At the start of the protocol, the estimator's weights (segmented rectangular box) are untrained (grey). The different segments separated by vertical lines correspond to the weights for distinct subsets of error components. At each iteration, distinct subsets of error components and their weights are isolated and trained on training data corresponding to judiciously chosen benchmarks. Isolated benchmarks isolate the relaxation, dephasing, and pulse distortion error components, while parallel benchmarks introduce stray coupling. Once trained, the weights are fixed to their trained values (blue) and the training protocol proceeds onto the next iteration. At the end of the protocol, all of the estimator's weights are trained.

(Supplementary Fig. 3). Early iterations train the estimator on isolated benchmarks, which isolate the weights corresponding to relaxation and dephasing. Later iterations train the estimator on parallel benchmarks, which introduce weights corresponding to stray coupling. This protocol was designed specifically to further suppress the capacity of the model trained at any given iteration.

We implement training iterations within the standard machine learning framework[6–8,37], using $\sim 60\%$ of benchmarks for training and the remaining $\sim 40\%$ for computing accuracy metrics. The error components are the training features and the error benchmarks are the training targets. At each iteration, we train using the Adam optimizer[38] with a mean-absolute-error cost function, which is less susceptible to outliers than the more standard mean-squared-error. Since the trained weights are not generally useful, they will not be presented.

4.4. Accuracy

We evaluate the accuracy of our trained estimator on the test data, which was not used during training. Our trained estimator can predict both SQRB and CZXEB benchmarks in arbitrary configurations. To evaluate the accuracy of these predictions, we consider the following metrics and quote the medians (Supplementary Fig. 4):

- Inaccuracy = |Predicted − Measured|

  Isolated SQRB $\sim 1.2 \times 10^{-4}$

  Parallel SQRB $\sim 1.6 \times 10^{-4}$

  Isolated CZXEB $\sim 1.4 \times 10^{-3}$

  Parallel CZXEB $\sim 1.9 \times 10^{-3}$

- Relative Inacc. = Inaccuracy/Measured

  Isolated SQRB $\sim 13\%$

  Parallel SQRB $\sim 20\%$

  Isolated CZXEB $\sim 22\%$

  Parallel CZXEB $\sim 27\%$

Although the medians are a concise statistic, they oversimplify the situation. Namely, inaccuracy metrics typically depend on and increase with the magnitude of benchmark values. These dependencies suggest that defining a trust region, within which we trust our estimator's predictions, may be more useful.

We define the trust region to be the range of Isolated and Parallel CZXEB test data over which the estimators inaccuracy is simultaneously $\lesssim 1/2\times$ the measured error and $\lesssim 2\times$ the experimental uncertainty (Supplementary Fig. 5). We interpret these as signal-to-noise metrics. Finally, we note that CZXEB benchmarks are the hardest to predict, requiring accurate SQ and CZ gate error predictions.

- Trust region: ~ 3 to $\sim 40 \times 10^{-3}$

Therefore, we can trust our estimator within a wide range that spans one order of magnitude.

4.5. Innacuracy sources

We expect our estimator's predictions to deviate from measurements for reasons including:

- Simplifications and/or approximations made to the error components to suppress runtimes.
- Less training data towards higher errors.
- Undiscovered physical error mechanisms.
- Control and hardware parameter inhomogeneities.

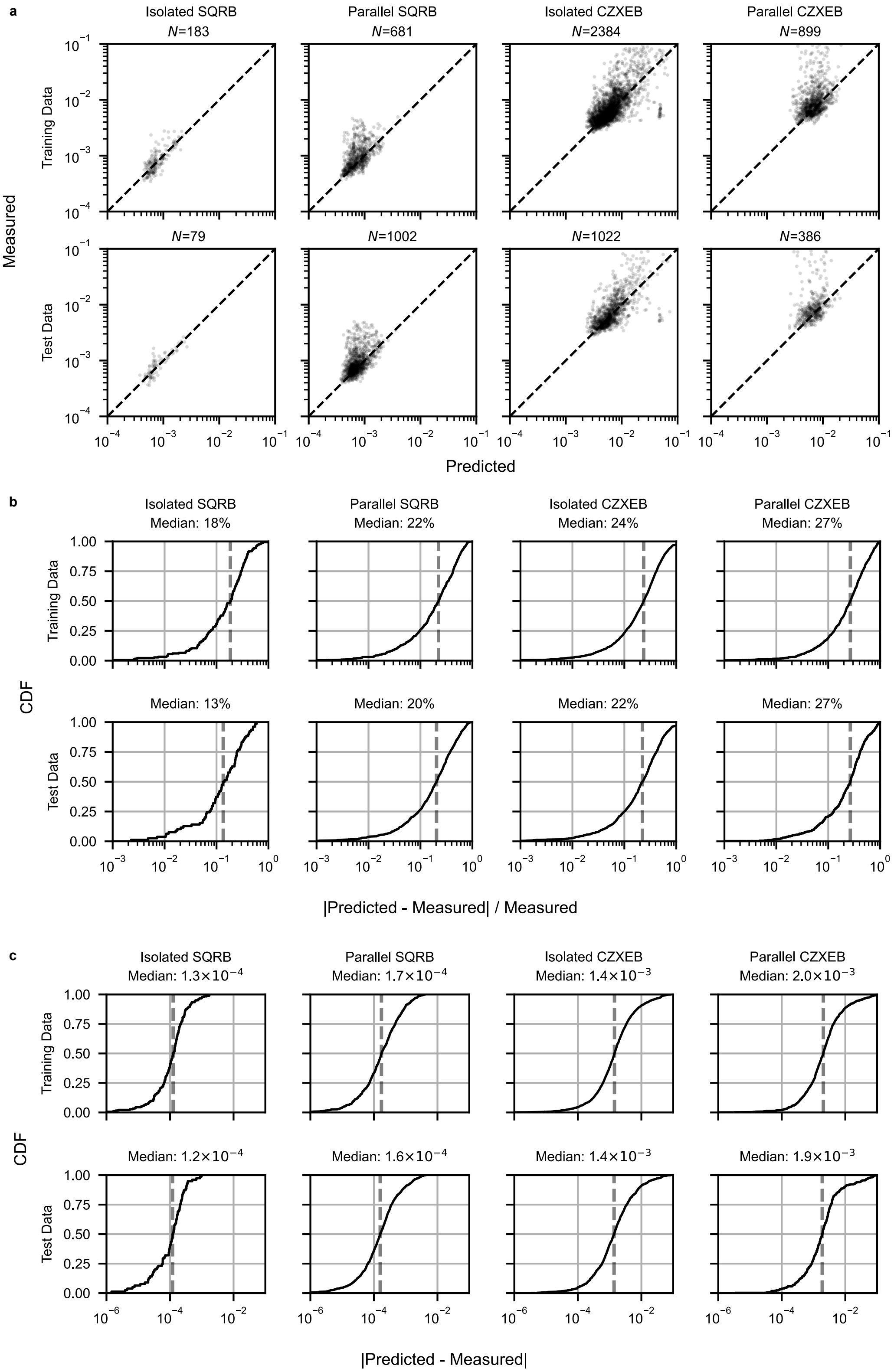

Supplementary Fig. 4. **Algorithm error estimator predictions versus measurements**. (a) Measured versus predicted SQRB (first two columns) and CZXEB (last two columns) benchmarks for the training (first row) and test (second row) data and for Isolated and Parallel benchmarks. N is the number of samples. The data were taken in frequency configurations of variable complexity. Deviations from the diagonal dashed line - where predictions match measurements - correspond to inaccuracies. (b) Cumulative distribution functions (CDF) of the Inaccuracy and (c) Relative Inaccuracy for the data in (a). The dashed vertical lines in (b) and (c) correspond to the distribution medians.

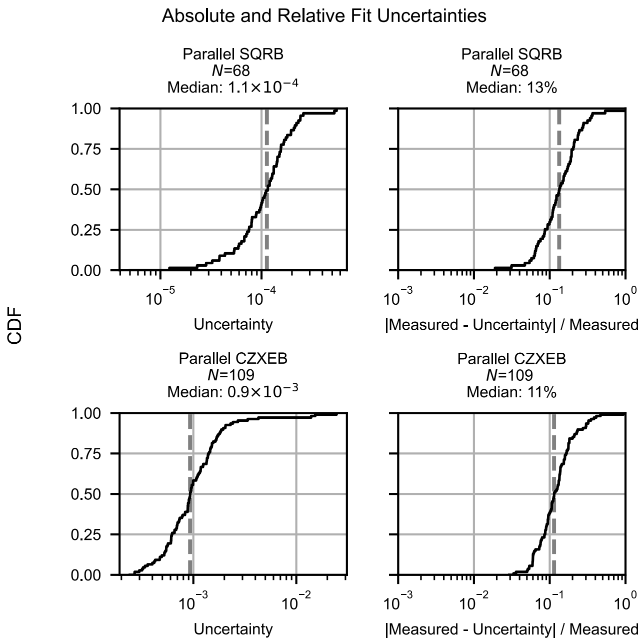

Supplementary Fig. 5. **Experimental uncertainty**. Experimental uncertainty and relative uncertainty for Parallel SQRB and Parallel CZXEB benchmarks for one 68 qubit configuration. The dashed vertical lines correspond to the distribution medians. N is the number of samples.

For further insights, we first inspect predicted versus measured data (Supplementary Fig. 4). The largest inaccuracies are seen towards high parallel CZXEB errors ($\sim 5\%$ of the training and test data). Since similar inaccuracies are not as prevalent in other benchmarks, we believe the primary culprits are:

- Inaccuracies in the CZ stray coupling error components, which are known to be complex.
- The impact of leakage, which can be driven via stray coupling, on CZXEB is not well understood.

Second, we inspect the isolated CZXEB data (Supplementary Fig. 6). Here the interaction frequencies are swept while idles are fixed. These data should be interpreted as line cuts of the processor's higher dimensional error landscape, where all idles and interactions are variables. Isolated CZXEB mostly isolates dephasing and relaxation errors, the latter of which dominate in our system and exhibit the most complex patterns. These data suggest that our estimator can reproduce complex error patterns, some of which span an order of magnitude and exceed the trust region defined above. However, we also observe clear inaccuracies, the most significant of which we believe are due to:

- TLS fluctuations[15,39] between characterization, optimization, calibration, and benchmarking.
- Underrepresented physics corresponding to qubits interacting with strongly-coupled TLS[16].

5. OPTIMIZATION SYSTEM

Our optimization system is based on the Snake optimizer, which we proposed[40,41] as a platform for deploying custom optimization strategies within the demands of an industrial control system. Snake leverages concepts in dynamic programming and graph optimization to offer several key functionalities, which to the best of our knowledge are not offered by any other optimizer:

- Flexibility: Can implement a wide array of optimization strategies via judicious selection of several parameters (Supplementary Note 5.3). Most notably, it can deploy virtually any inner loop optimizer at any dimension between the local and global optimization limits. This customizability facilitates adapting Snake to a variety of optimization landscapes.
- Scalability: Runtime scales linearly versus processor size without parallelization and sub-linearly with geometric parallelization (stitching).
- Stability: Can locally re-optimize performance outliers (healing) as they emerge over time to stabilize processors over long timescales (i.e. months). Healing is much faster than optimizing a full processor (Supplementary Note 5.5) and scales sub-linearly with the number of outliers when parallelized.

In our past proposal, we established Snake's theoretical foundations and software abstractions. Here we map frequency optimization into Snake. We then provide broadly applicable implementations of its parameters and employ them to explore tens of optimization strategies between the local and global optimization limits over thousands of simulated optimization threads. Finally, we promote the most promising strategies to the experiments in the manuscript.

5.1. Optimization variables

Qubit frequency trajectories can be parameterized by a variety of optimization variables. We allocate one variable per gate:

- Idle frequency (f_i): The $|0\rangle \leftrightarrow |1\rangle$ transition frequency where q_i executes SQ_i.
- Interaction frequency (f_{ij}): The average of the uncoupled $|0\rangle \leftrightarrow |1\rangle$ transition frequencies, where q_i and q_j execute CZ_{ij}.

We choose these variables because we have empirically verified that they have a significant impact on gate performance. Although we could add more trajectory variables, for example gate lengths, each such variable exponentially expands the configuration space and presents a scalability barrier. Instead we either fix or locally optimize those variables within our calibration system[1].

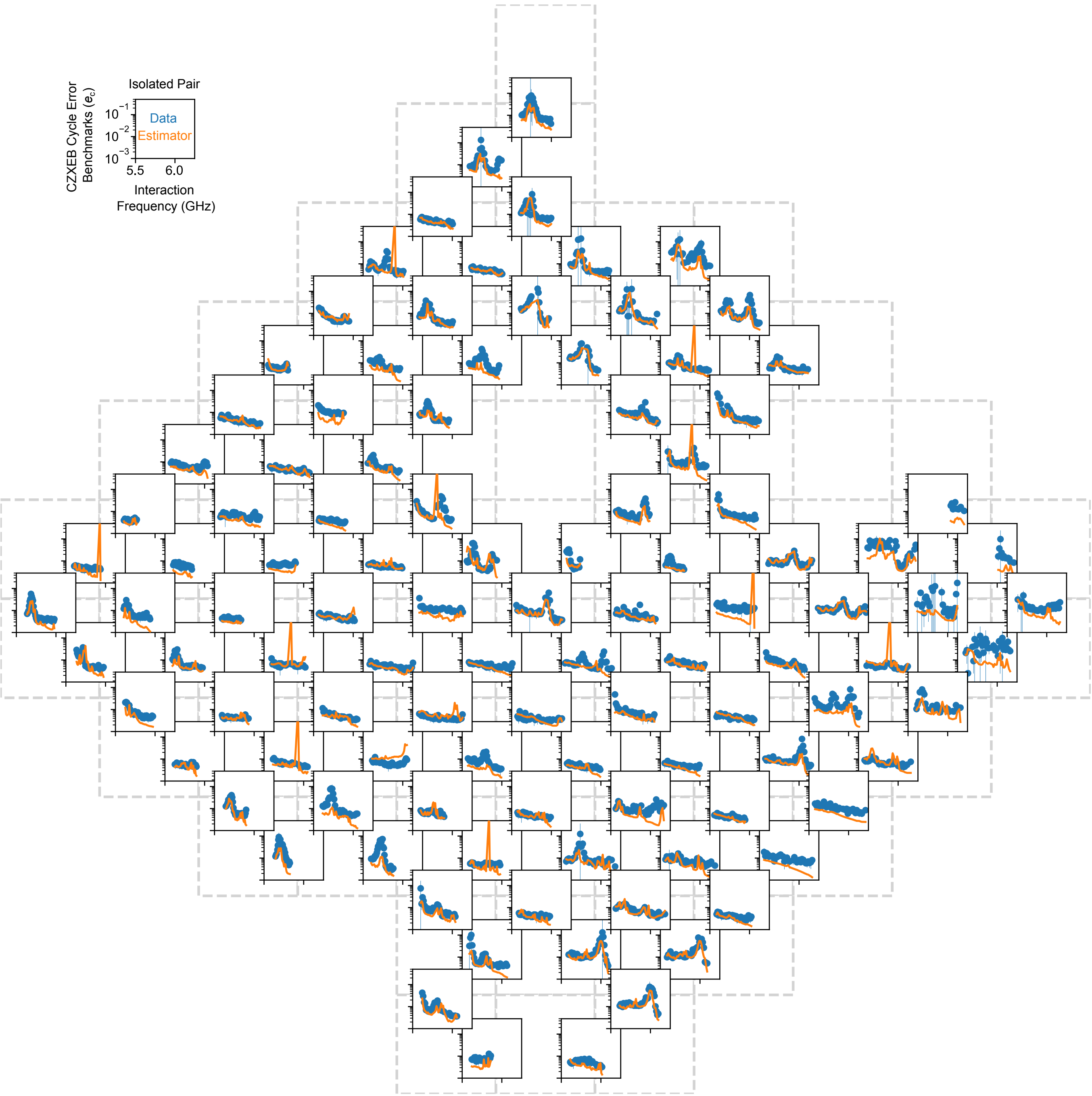

Supplementary Fig. 6. **Isolated CZXEB benchmarks versus interaction frequency**. These data are taken by fixing idle frequencies and benchmarking CZXEB at various interaction frequencies. They can be interpreted as linecuts of the processors much higher dimensional error landscape, where all idles and interactions are variables. The inset shows the common scale, with the data (blue) and algorithm error estimator predictions (orange) overlaid. Error bars correspond to 68% confidence intervals and are typically smaller than the data points. The error axis (vertical) is logarithmically spaced to highlight small inaccuracies. The dashed boxes (grey) represent the qubits of our processor and map to the processor graph in Supplementary Fig. 1b. The algorithm error estimator can reproduce complex error patterns, some of which span an order of magnitude. However, there are also clear inaccuracies, some of which are discussed in Supplementary Note 4.5.

5.2. Optimization bounds

Each frequency variable is subject to hard bounds set by the quantum and classical control hardware. The bounds are computed from the characterization data and embed metrology of our hardware and control systems. Furthermore, they are contracted as much as reasonably possible via physics intuition to reduce the configuration space and thus optimization runtimes. Here we list several hard bounds:

- Idle frequency bounds

  Maximum detuning from qubits' maximum frequencies to limit dephasing.

  Minimum detuning from qubits' readout resonators to limit Purcell relaxation and resonator-induced dephasing[22,42].

  Minimum and maximum detuning from the LO to limit microwave pulse-distortion due to the finite DAC bandwidth.

- Interaction frequency bounds

  Maximum detuning from qubits' maximum frequencies to limit dephasing.

  Minimum detuning from qubits' readout resonators to limit Purcell relaxation and resonator-induced dephasing[22,42].

For $N = 68$ configurations, these hard bounds constrain SQ and CZ gates to average operating bandwidths $\sim 450 \pm 120$ ($\pm 1\sigma$) MHz and $\sim 635 \pm 112$ MHz, respectively. From these operating bandwidths, together with a 2 MHz hardware discretization, we can estimate the average number of idle and interaction frequency options (k in the manuscript) and the total number of frequency configurations for the processor:

- Idle frequency options: $\sim 225 \pm 60$.
- Interaction frequency options: $\sim 318 \pm 56$.
- Frequency configurations: $\sim 2^{1437}$ total configurations for our processor. This number significantly exceeds our processor's 2^{68} Hilbert space dimension, and even the $\sim 2^{265}$ atoms in the visible universe, which is surpassed near $N \sim 10$.

5.3. Optimization parameters

Snake can implement a wide array of optimization strategies through judicious selection of four parameters - the seed strategy, traversal strategy, scope, and inner-loop optimizer. These must be tuned to the problem of interest, while balancing runtime and performance, which often compete. We interpret this tuning as adapting Snake to the optimization landscape presented by the algorithm error estimator and expect it to be especially critical when applying our strategy beyond our hardware.

Below we overview each parameter's role and then tune them over $\sim 7,000$ simulated optimization threads of our processor. We evaluate their runtime and performance against an untrained but representative algorithm error estimator, quoting relative performance measures (Supplementary Fig. 7). By tuning the parameters against an estimator and not hardware benchmarks, these results are not susceptible to drift or the estimator's accuracy in predicting benchmarks. Furthermore, an experiment of this scale would take ~ 1.5 years in hardware.

Once tuned, the parameters are remarkably robust. We have seen one set of tuned parameters remain reasonably effective for a variety of quantum algorithms and multiple generations of frequency tunable qubits, some of which underwent significant architectural modifications.

5.3.3. Seed strategy

Determines how Snake prioritizes seed gates to launch optimization threads from.

- Performance impact: $\sim 15\%$ variations.
- Runtime impact: Negligible variations.
- Tuned value: For experimental configurations up to $N = 68$, we generated solutions from all seeds and selected the one that minimized the trained algorithm error estimator. For larger simulated processors, we selected a subset of all seeds randomly.

We note that when scaling beyond hundreds of qubits, generating solutions from all seeds will become prohibitively time consuming. Identifying a strategy for seeding gates that may offer a performance advantage is thus expected to become important. One systematic approach for developing such a strategy is to correlate optimized estimator values against statistics computed from the corresponding seeds characterization data, which are available prior to optimization.

5.3.3. Traversal strategy

Determines how Snake drives graph traversal within each optimization thread, which we characterize via a traversal rule and traversal heuristic:

- Traversal rule: Identifies candidate gates for traversal, given the current gate. Here we considered nearest-neighbor (NN), next-nearest-neighbor (NNN), and arbitrary-scope (ARB) rules. ARB is a generalization of the NN and NNN rules that identifies candidates within an annular ring whose radius is set by the scope parameter. It was developed for $S > 3$ optimization, where NN and NNN traversals are too short.

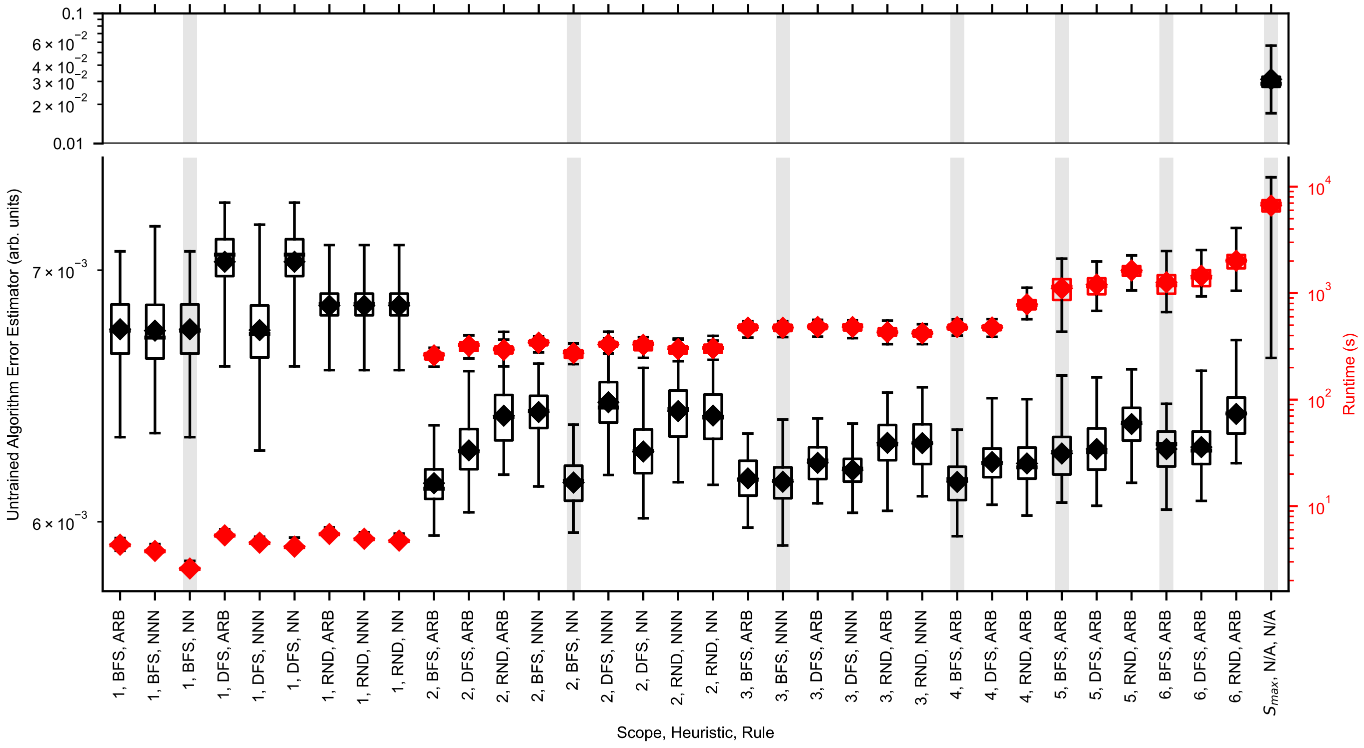

Supplementary Fig. 7. **Tuning Snake's parameters**. The optimized untrained algorithm estimator value (black boxes) and runtime (red boxes) corresponding to $N = 68$ optimization threads versus Snake's scope, traversal heuristic, and traversal rule parameters. Each box corresponds to multiple seeds and shows the respective distribution's 0, 25, 50, 75, and 100th percentile (horizontal notches) and mean (diamond). The gray vertical bars are the tuned parameters used in experiment for each scope.

- Traversal heuristic: Sorts candidate gates returned by the traversal rule to implement some desired traversal pattern. Here we tested textbook breadth-first (BFS), depth-first (DFS), and random (RND) heuristics[43].

- Performance impact: $\sim 15\%$ variations.

- Runtime impact: Negligible variations.

- Tuned value: We use a scope-dependent traversal strategy (gray bars in Supplementary Fig. 7). However, the ARB traversal rule with the BFS traversal heuristic often outperformed other strategies.

We note that many algorithms exist for approaching graph-based problems[7,43] and can be adapted to Snake. For example, heuristics such as the fail-first heuristic (i.e. prioritize traversals between highly constrained gates) and/or techniques such as back-tracking[7] and/or Monte Carlo Tree Search (MCTS)[7,44] may offer advantages beyond the textbook strategies tested. Multiple optimization traversals, which we consider an extension of healing, may also boost performance.

5.3.3. Scope

Bounds the dimension of the optimization problem solved at each traversal step. We note that we refer to S as the distance d_P in reference 41. We do not adopt that name here to avoid collisions with the error correction distance d. Here we tested scopes $S = 1, 2, 3, 4, 5, 6, S_{\mathrm{max}}$, which correspond to maximum optimization dimensions of 1D (local), 5D, 9D, 21D, 29D, 49D, $|F|$D (global).

- Performance impact: $\sim 5\times$ variations.

- Runtime impact: $\sim 10^4\times$ variations.

- Tuned value: $S = 4$ ($\leq$ 21D) found the lowest estimator values, when averaged over all tested parameters. However, $S = 2$ ($\leq$ 5D) offers a better balance between performance (only $\sim 2\%$ worse) and runtime ($\sim 50\%$ faster) and is our tuned value.

We note that the scope may be dynamically varied at each traversal step depending on the characterization data. For example, larger scopes may outperform lower scopes when optimizing highly constrained gates with anomalous circuit parameters and/or particularly detrimental TLS defects[15]. Furthermore, we expect the optimal scope to depend strongly on the hardware architecture and to scale with the spatial extent of engineered and

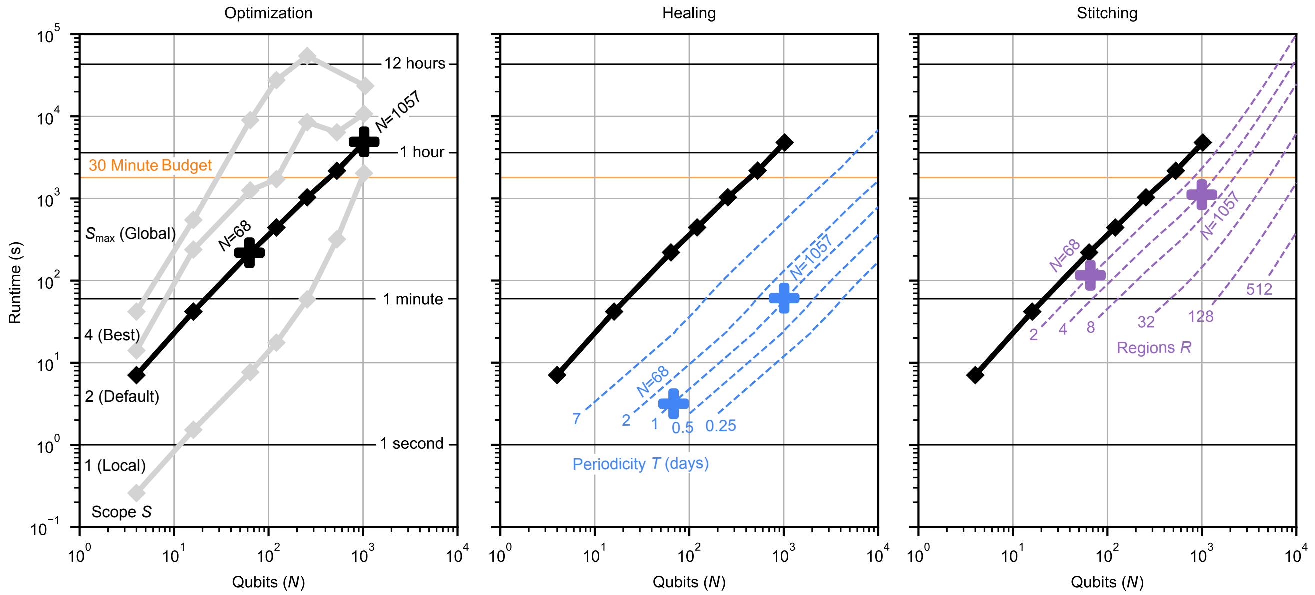

Supplementary Fig. 8. Optimization, healing, and stitching runtime scalability. (a) Optimization runtime versus simulated processor size N for several scopes S of interest (grey and black curves). The default scope $S = 2$ (black) and 30 minute optimization budget (orange) are reproduced on all panels. The + markers are points of interest for the largest experimental ($N = 68$) and simulated ($N = 1057$) configurations investigated. (b) Estimated healing runtimes (blue curves) for several heal periodicities T. The + markers correspond to daily healing. We extrapolate runtimes below the range of available data. (c) Estimated stitching runtimes (purple curves) for several stitched regions R. The + markers correspond to the stitching experiment and simulation in Fig. 4.

parasitic interactions. Optimizing hardware with higher connectivity (e.g. with three-qubit gates) would likely benefit from a larger scope than lower connectivity (e.g. with two-qubit gates). Similarly, optimizing hardware with longer-range stray coupling would benefit from a larger scope than shorter-range stray coupling.

5.3.3. Inner loop optimizer

Determines how Snake optimizes the Snake estimator at each traversal step. Here we considered differential evolution[45], L-BFGS[46], simplical homology global optimization[47], dual annealing[48], dividing rectangles[49], and basin hopping[50].

- Performance impact: Significant variations.
- Runtime impact: Significant variations.
- Tuned value: We use a dimension-dependent inner-loop optimizer. We exhaustively search < 3D problems for their globally optimal values. We stochastically search ≥ 3D problems via a tuned global optimizer.

We note that Snake can deploy nearly-arbitrary continuous or discrete inner-loop optimizers, treating the optimization variables accordingly. Of particular interest are model-free reinforcement-learning agents[7,44,51,52]. In the short term, model-free agents could refine configurations found via model-based optimization to compensate for inaccuracies in the algorithm error estimator, which are expected to increase with processor size due to increased control and hardware inhomogeneities. In the longer term, they could replace model-based optimization entirely[53,54] and eliminate the research burden of developing performance estimators.

5.4. Optimization runtime budget

We develop a runtime budget with the objective of operating a distance 23 logical qubit with $N = 1057$ physical qubits. Since the surface code has a lenient qubit failure tolerance[55] of $> 1\%$ and since our outlier emergence probability is ~ 0.01 / 24 hours / gate (Supplementary Note 8.2), we have $\sim$ 24 hours to characterize, optimize, calibrate, and benchmark our processor and finally execute the algorithm before restarting the process.

If we target 12 hours for executing the algorithm, we have 12 hours to go from characterization to benchmarking. Since characterization, calibration, and benchmarking take $\sim$ 2 hours and are nominally independent of processor size due to parallelization, we have $\sim$ 10 hours left. From this large window, we only budget 0.5 hours for optimization, leaving $\sim$ 9.5 hours for unforeseen scaling overhead.

The 0.5 hour runtime budget can be fulfilled via a com-

bination of optimization, healing, and stitching (Supplementary Note 5.5). As the hardware evolves, we expect the outlier emergence probability to decrease and the control system to become faster through hardware and software advancements, which should relax all budgets and enable operating even larger surface codes.

5.5. Optimization runtime scalability

To understand how optimization thread runtimes scale with processor size, we optimize simulated processors of variable size at several scopes (Supplementary Fig. 8). The trends are complex, but runtimes generally increase with both scope and processor size. For our default scope $S = 2$ in particular, runtimes fit well to the heuristic scaling model $r = a + bN + cN^2$, where r is the runtime, N is the number of qubits, and a, b, and c are heuristic coefficients.

Fitting this model to our data, we find best-fit parameters $a = -8.4 \pm 15.5$ seconds, $b = 3.6 \pm 0.1$ seconds / qubit, and $c = 0.0010 \pm 0.0001$ seconds / qubit^2. The linear term dominates towards hundreds of qubits, as we would expect from Snake's algorithmic structure. This is the term quoted in the performance scalability section of the manuscript.

To estimate healing runtimes r_h, we assume that outliers emerge with probability 0.01 / 24 hours / gate (Supplementary Note 8.2), that each outlier conservatively targets 2 qubits' idle and interaction frequencies for healing (Supplementary Note 8.3), and that healing occurs periodically every T days. In combination, $r_h = 0.01 \times 2 \times T \times r$.

To estimate stitching runtimes r_s, we assume that a processor can be split into approximately equal-sized regions and that stitching overhead is negligible. We believe the latter assumption is valid, especially since stitching can itself be parallelized. In combination, $r_s = r/R$.

6. BENCHMARKING SYSTEM

The benchmarking system measures the performance of frequency configurations via benchmarking algorithms that should be representative of the target quantum algorithm but much cheaper to execute (Supplementary Fig. 9). Here we use:

- Two-qubit cross-entropy benchmarking (CZXEB): Measures the error per cycle $e_{c,ij}$ for SQ_i, SQ_j and CZ_{ij}, respectively[1,5]. Four CZXEB benchmarking algorithms with distinct CZ layer patterns are necessary to benchmark all CZ gates (Supplementary Fig. 9c). They are run back-to-back during benchmarking. When referencing CZXEB, we reference all of these algorithms and their respective benchmarks in combination.

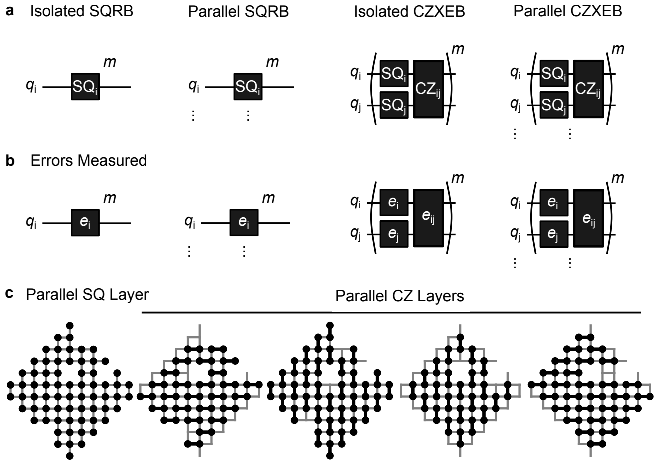

Supplementary Fig. 9. **Performance benchmarks**. (a) Benchmark types used to evaluate performance. The ellipses indicate more qubits and respective gates benchmarked in parallel. Initialization into $|0\rangle$ and measurement in the Z basis are implicit. (b) Errors measured by the benchmark types in (a). (c) SQ and CZ gate layers used for the parallel benchmarks in (a). Measuring CZXEB for all gates requires running four distinct algorithms, each of which is characterized by interleaving a distinct CZ layer with the SQ layer. When referencing CZXEB, we implicitly reference all four algorithms and corresponding benchmarks.

- Single-qubit randomized benchmarking (SQRB)[56]: Measures the error per gate e_i for SQ_i. These benchmarks were not presented in the manuscript since CZXEB is considered a more holistic metric. SQRB benchmarks corresponding to the experiments in the manuscript are in Supplementary Figs. 10 - 12. SQRB can be combined with CZXEB to infer CZ error contributions to the cycle errors ($e_{c,ij}$ for CZ_{ij}).

In some cases, we label benchmarks as follows:

- Isolated: Benchmarks taken in sparse configurations where stray coupling is negligible.
- Parallel: Benchmarks taken in denser configurations where stray coupling is non-negligible.

All SQRB and CZXEB benchmarks are taken in parallel, unless otherwise noted, and reported as average errors. Conversion factors between average errors, depolarizing errors, and Pauli errors are defined in Table I of Section V of the Supplementary information of Reference 1. Statistics for SQRB and CZXEB distributions presented in Figs. 2 - 4 and Supplementary Figs. 10 - 12 are in Supplementary Tables 1 - 4.

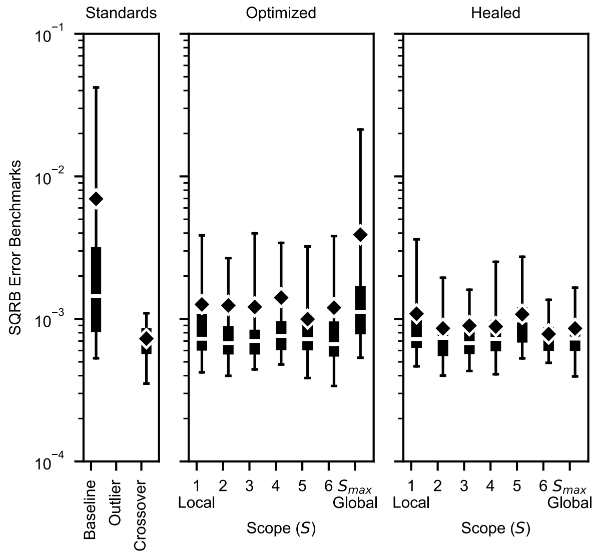

Supplementary Fig. 10. **Optimization and healing performance**. SQRB benchmark distributions corresponding to the configurations presented in Fig. 2. An outlier standard is not defined for SQRB. SQRB trends are consistent with CZXEB.

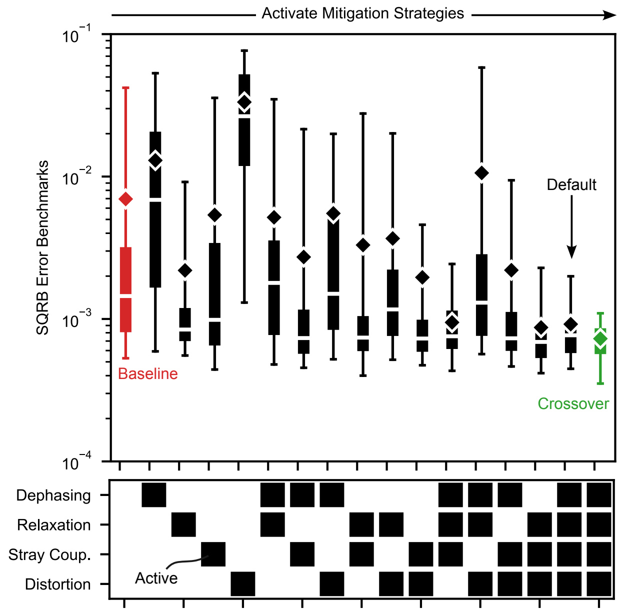

Supplementary Fig. 11. **Optimization performance for various error mitigation strategies**. SQRB benchmark distributions corresponding to the configurations presented in Fig. 3. An outlier standard is not defined for SQRB. SQRB trends are consistent with CZXEB.

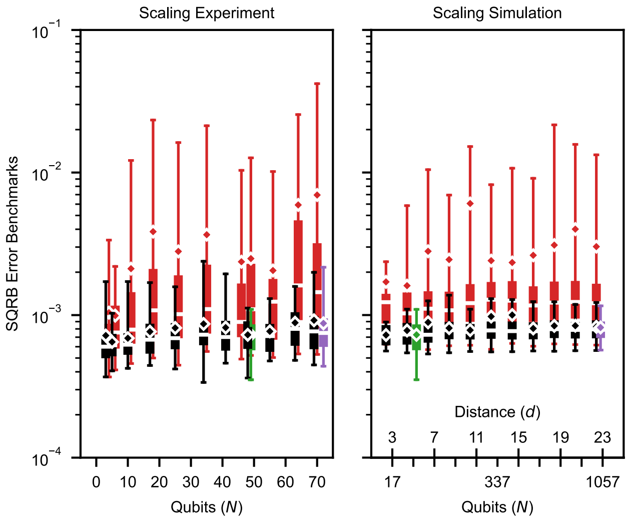

Supplementary Fig. 12. **Optimization scalability**. SQRB benchmark distributions corresponding to the configurations presented in Fig. 4. An outlier standard is not defined for SQRB. SQRB trends are consistent with CZXEB. Some boxes have been horizontally shifted to reduce overlap.

7. SIMULATION ENVIRONMENT

Due to complex interplay between hardware inhomogeneities, error components, hard bounds, and our control optimization strategy, trustworthy scaling simulations require us to emulate our quantum computing stack from the hardware to the control system. We emulate them as follows:

- Hardware: We developed[35] a generative model of our quantum processor architecture (more below). This approach enables us to embed the statistics of empirically measured characterization data into our simulations.
- Characterization: We sample[7] the generative model to generate simulated processor characterization data. Each random sample is distinct but should nominally follow the statistics of empirically measured characterization data.
- Optimization: We use the Snake optimizer on simulated characterization data exactly as we would on real characterization data.
- Calibration and Benchmarking: We use our trained algorithm error estimator on optimized configurations with the simulated characterization data to estimate benchmarks. We assume that the impact of calibration is implicitly embedded into the trained weights of the estimator.

Since the generative model is an important component of the simulation environment and has not yet been discussed in detail, we provide an overview below and direct the reader to Reference 35 for details.

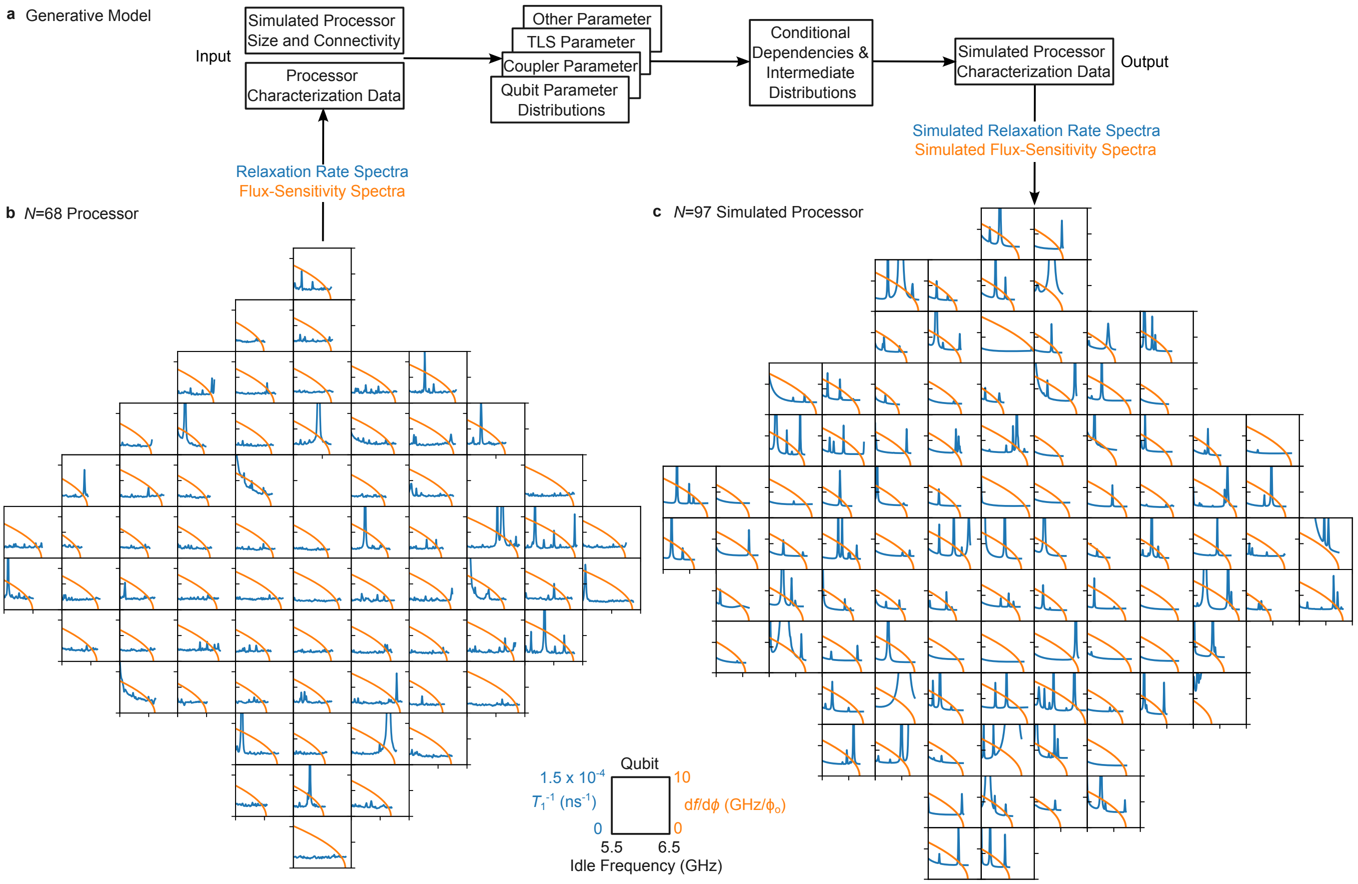

Supplementary Fig. 13. **Generating simulated processors**. (a) Schematic of the generative model that we use to generate simulated processor characterization data. (b) Comparison of the energy relaxation rate (T_1^{-1}, blue) and flux-sensitivity spectra ($\frac{df}{d\phi}$, orange) for our $N = 68$ processor and (c) $N = 97$ simulated processor. The inset shows the common scale. Our generative model produces simulated processor characterization data that are nearly indistinguishable from our processor.

7.1. Simulated processor generative model

Our goal is to develop a statistical model that can be sampled for characterization data for simulated processors of arbitrary size and connectivity that are statistically indistinguishable from our real quantum processor. Towards that end, we interpret an arbitrary quantum processor as a statistical sample from some quantum processor joint probability density $P(D, \mathcal{P}, \mathcal{N})$. Here D is the characterization data, $\mathcal{P}$ is a set of architectural parameters (e.g. qubit circuit parameters, coupler circuit parameters, TLS parameters), and $\mathcal{N}$ is the processor's size and connectivity. Within this statistical picture, $D, \mathcal{P}, \mathcal{N}$ are random variables.

Generating a simulated quantum processor and its corresponding characterization data amounts to sampling P. To do so, we consider the chain-rule decomposition $P(D, \mathcal{P}, \mathcal{N}) = P(D|\mathcal{P}, \mathcal{N})P(\mathcal{P}|\mathcal{N})P(\mathcal{N})$, which can be represented as a Bayesian network, and employ prior sampling[7] (Supplementary Fig. 13a) as follows:

1. Select simulated processor size and connectivity $\mathcal{N}$.

2. Sample architectural parameters for all qubits of the simulated processor from $P(\mathcal{P}|\mathcal{N})$ under the naive assumption that they are conditionally independent amongst themselves and the qubits. The distributions of architectural parameters were determined by statistically analyzing our real quantum processor's characterization data.

3. Sample characterization data by propagating the architectural parameters through conditional dependencies $P(D|\mathcal{P}, \mathcal{N})$ determined through textbook physics, published literature, and metrology.

If the generative model is accurate, the simulated processor's characterization data should be statistically indistinguishable from our real quantum processor.

7.2. Simulated characterization data

We validate the accuracy of our generative model by comparing relaxation and flux-sensitivity spectra sampled for a simulated processor against our real processor (Supplementary Fig. 13b-c). We believe this is a holistic test of accuracy because these spectra have complex characteristics that require an accurate confluence of architectural parameters to accurately reproduce. Apart from experimental noise, which are filtered during optimization, the simulated spectra are nearly indistinguishable from real spectra. In turn, we believe the generative model is sufficiently accurate for trustworthy simulations.

8. ADDITIONAL EXPERIMENTAL DETAILS

8.1. Experimental controls

Due to the complexity of our quantum computing stack, developing good experimental controls for any subcomponent - including our optimization system - is nontrivial. Our primary controls are the random baseline configurations. These are expected to sample the average performance of the hardware and calibration systems without frequency optimization. However, we note that all baseline configurations are generated within frequency hard bounds, which themselves embed some error mitigation (Supplementary Note 5.2). Therefore, we believe the performance advantage quoted in the manuscript for our optimization strategy is an underestimate.

8.2. Impact of drift

Both slow gradual drift (e.g. due to temperature fluctuations in the lab) and abrupt catastrophic drift (e.g. due to TLS fluctuating into gates) can generate outliers over time. To understand the extent to which drift impacts our experimental results, we compare the runtimes of the components of our control system against the estimated rate at which outliers emerge for $N = 68$ configurations.

- Characterization: ~ 0.5 hours / experiment.
- Optimization: 0.5 hours budgeted / experiment.
- Calibration: ~ 1 hours / experiment.
- Benchmarking: $\sim$ 0.2 hours / experiment.
- Total runtime: ~ 2.5 hours / experiment.
- Outlier emergence probability: ~ 0.01 / 24 hours / gate.

If we naively assume that outliers are conditionally independent and binomially distributed, we expect drift to generate:

$$\sim 177 \text{ gates} \times \frac{0.01}{24 \text{ hours} \times \text{gate}} \times \frac{2.5 \text{ hours}}{\text{experiment}} \sim \frac{0.2 \text{ outliers}}{\text{experiment}}$$

We thus do not believe that our results are significantly impacted by outliers emerging from drift.

8.3. Healing experiment

For the healing experiment presented in Fig. 2, frequencies were targeted for healing via thresholding and manual discretion as follows:

- Interaction frequencies f_{ij} were typically targeted when their corresponding CZXEB benchmarks exceeded the outlier standard ($e_{c,ij} \geq 1.5 \times 10^{-2}$). Re-optimizing interactions is relatively inexpensive and does not necessarily require the corresponding idles to be re-optimized.
- Idle frequencies f_i were typically targeted when their corresponding SQRB benchmarks were anomalously high ($e_i \gtrsim 1.5 \times 10^{-3}$) and/or when they corresponded to multiple CZXEB benchmarks that exceeded the outlier standard. Re-optimizing idles is relatively expensive and requires all hinged interactions to be re-optimized also.

Heals were conducted within ~ 1 hour of benchmarking the optimized-but-unhealed configurations to mitigate the impact of drift. Furthermore, new characterization data were taken for targetted devices under the assumption that the original data were no longer valid. The primary concerns are TLS fluctuations[15,39], which are represented in the T_1 spectra.

8.4. Metrology experiment

In Supplementary Fig. 14 , we show frequencies and cycle errors for all 16 configurations considered in the metrology experiment in Fig. 3, each of which was optimized with distinct error mitigation strategies active. In the following sections, we qualitatively and quantitatively analyze these data to understand the impact of each error mitigation strategy and their interplay.

8.4.4. Qualitative analysis

To qualitatively understand the impact of and interplay between error mitigation strategies, we inspect CZXEB cycle error contributions e_m for all error mechanisms m across all 16 configurations, each of which corresponds to distinct error mitigation strategies s active (Supplementary Fig. 15). Here $e_m = \langle e_{m,ij} \rangle_{ij}$, where $\langle . \rangle_{ij}$ is the median over pairs ij. The median was chosen

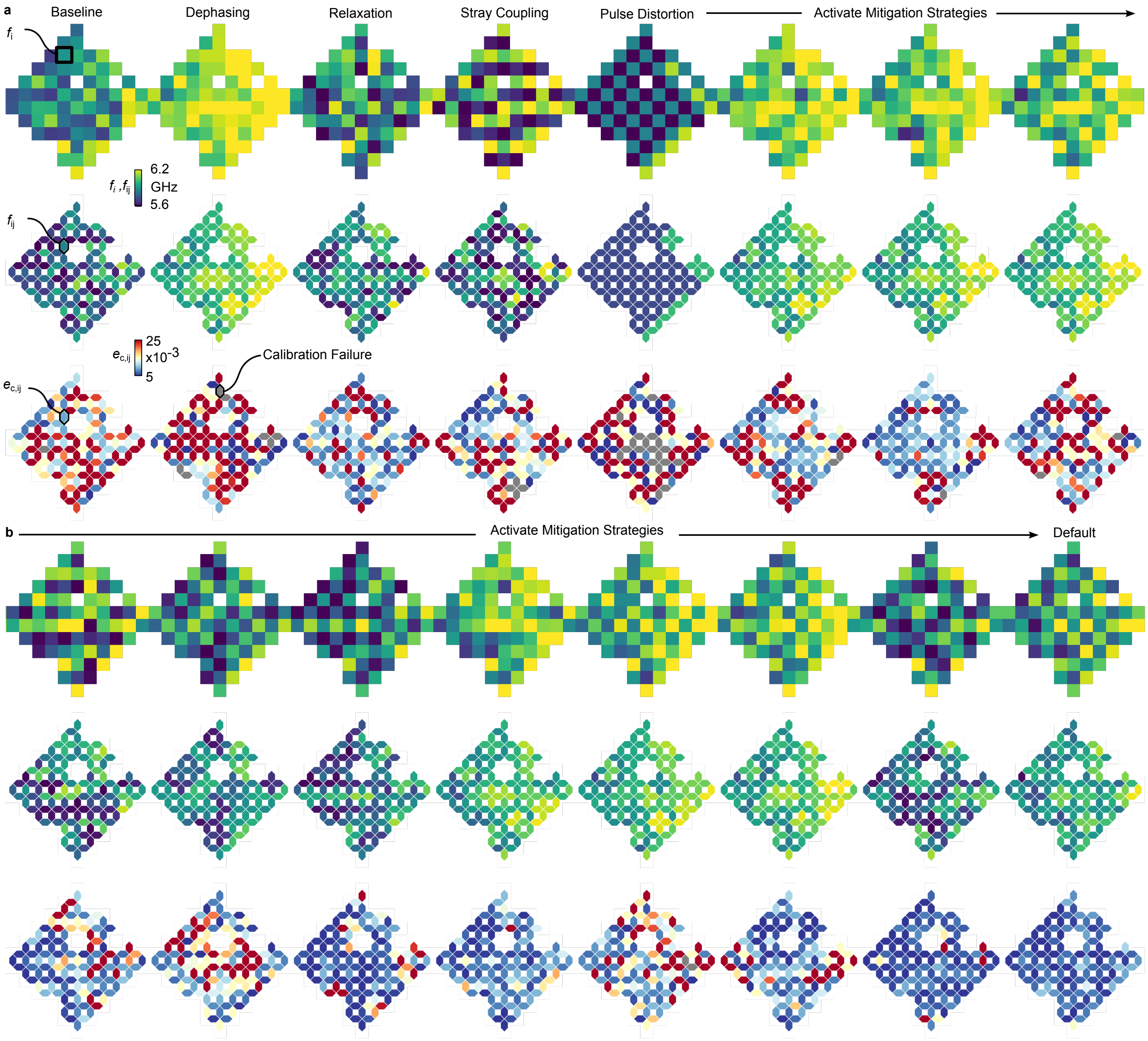

Supplementary Fig. 14. **Error metrology frequency configurations and benchmarks**. (a) Idle frequencies (f_i), interaction frequencies (f_{ij}), and CZXEB cycle errors ($e_{c,ij}$) for configurations optimized with all combinations of error mitigation strategies activated, to supplement the data shown in Fig. 3. (b) Continuation of (a), with all annotations shared.

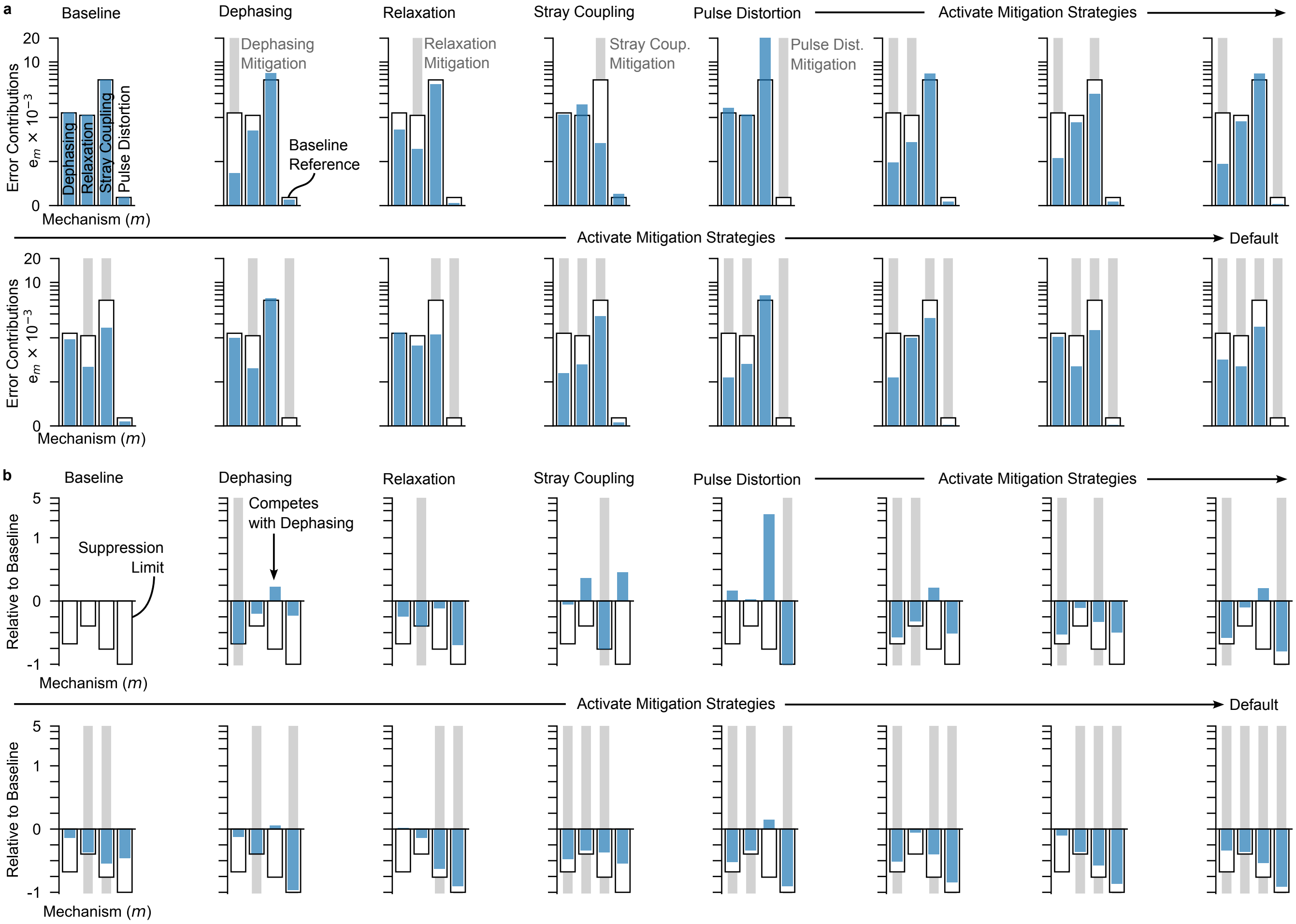

Supplementary Fig. 15. **Error metrology qualitative analysis**. (a) CZXEB cycle error contributions e_m for each error mechanism m and each configuration shown in Supplementary Fig. 14. Each bar corresponds to one error mechanism and each panel corresponds to a distinct combination of error mitigation strategies active (grey). The baseline is reproduced on each panel for reference (white). (b) Error contributions from (a) relative to the baseline to highlight interactions between mitigation strategies. Amplification in an error mechanism when another mitigation strategy is active is interpreted as competition. For example, we show in the second panel that dephasing competes with stray coupling. Each error mechanism's suppression limit, which is achieved when only its respective mitigation strategy is active, is reproduced on each panel for reference (white). Ideally, our optimization strategy would reach each error mechanism's suppression limit when all mitigation strategies are active (last panel).

to expose typical trends while preventing outliers from biasing our analysis. Each contribution $e_{m,ij}$ sums the corresponding weighted error components (e.g. SQ_i dephasing, SQ_j dephasing, and CZ_{ij} dephasing for $m =$ dephasing) as computed from the corresponding configuration's optimized frequencies and characterization data.

All panels in Supplementary Fig. 15 suggest that activating a particular error mitigation strategy suppresses its corresponding error mechanism as intended. However, the panels with only one mitigation strategy active also highlight non-trivial interactions between them. Of particular interest are amplifications in an error mechanism when another mitigation strategy is active, which we interpret as competition. Next we describe noteworthy competition and how it arises from the underlying physics.

Dephasing mitigation squeezes frequencies into a narrow band near their respective maxima where flux-sensitivity vanishes[22] $\frac{df}{d\phi} = 0$ (orange curves in Supplementary Fig. 13b). In turn, it boosts frequency collisions and thus competes with stray coupling. Stray coupling mitigation disperses frequencies to reduce frequency collisions. In turn, it boosts frequency excursions during CZs and thus competes with pulse distortion. Pulse distortion mitigation biases idles into $|11\rangle \leftrightarrow |02\rangle$ resonance (a checkerboard with neighbors q_i and q_j at $f_i = f_j - |\eta_j|$ or $f_j = f_i - |\eta_i|$, where η_i and η_j are qubit anharmonicities[2,57]) and interactions into resonance (at $f_{ij} = (f_i + f_j)/2$) to minimize frequency excursions during CZ gates. In turn, it boosts frequency collisions and thus competes with stray coupling. Finally, relaxation mitigation interacts non-trivially with all mecha-

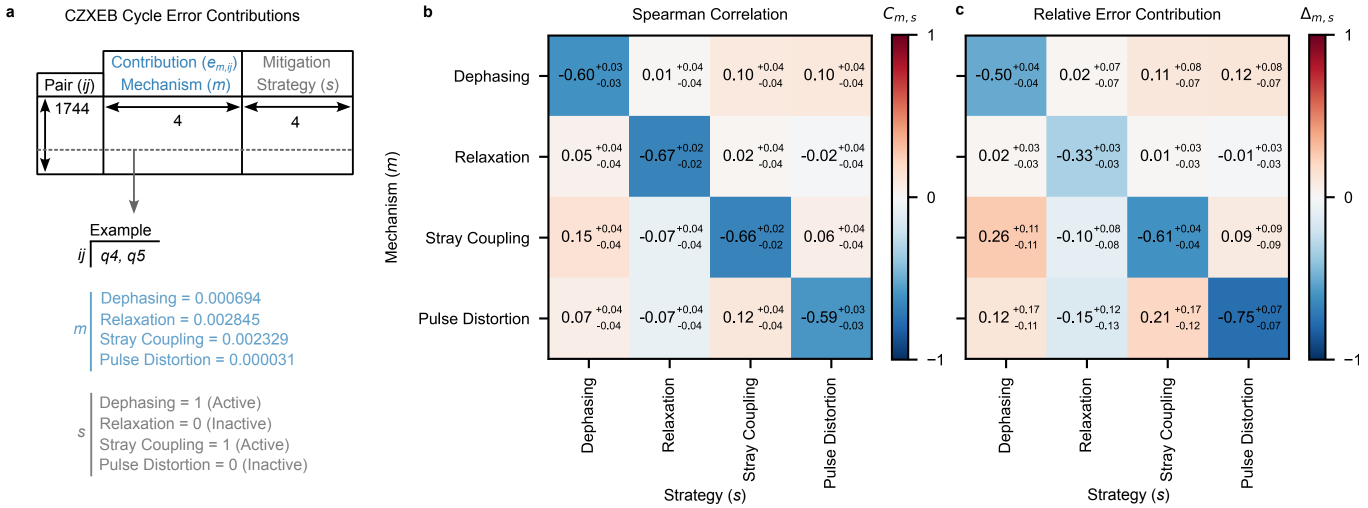

Supplementary Fig. 16. Error metrology quantitative analysis. (a) CZXEB cycle error contributions $e_{m,ij}$ for all error mechanisms m and all pairs ij across all 16 configurations, each of which corresponds to distinct error mitigation strategies s active. One row is shown for example. (b) Spearman correlation $C_{m,s}$ between the activation of error mitigation strategy s and error contributions from mechanism m. $C_{m,s} = -1$ means that activating strategy s perfectly monotonically suppresses error mechanism m. Ideally, all diagonals would be -1 and all off-diagonals would be 0. (c) Relative error contribution $\Delta_{m,s}$ from mechanism m when strategy s is active. $D_{m,s} = -1$ means that activating strategy s fully suppresses mechanism m. Ideally, all diagonals would be -1 and all off-diagonals would be 0. For (b) and (c) we report medians and their 95% confidence intervals.

nisms since it avoids relaxation hotspots with complex frequency dependencies[22,42,58,59] and randomness due to TLS[15] (blue curves in Supplementary Fig. 13b).

Despite nontrivial interactions between error mitigation strategies, the last panel of Supplementary Fig. 15b suggests that our optimizer can effectively reconcile their competition and suppress all error mechanisms simultaneously.

8.4.4. Quantitative Analysis

To quantitatively understand the impact of and interplay between error mitigation strategies, we analyze CZXEB cycle error contributions $e_{m,ij}$ for all error mechanisms m and all pairs ij across all 16 configurations, each of which corresponds to distinct error mitigation strategies s active (Supplementary Fig. 16a). Towards that end, we construct a database of error contributions with 1744 rows (109 pairs × 16 configurations) and 16 columns (4 mechanisms × 4 strategies), randomly sample ~ 500 rows, compute a metric of interest, and repeat 100,000 times. This bootstrapping procedure[6,8] generates a distribution for the metric, from which we report the median and 95% confidence interval.

Our first metric is the Spearman correlation coefficient ($C_{m,s}$) between each error mitigation strategy and mechanism (Supplementary Fig. 16b). The Spearman correlation identifies monotonic trends, making it more suitable for analyzing non-linear data with outliers than the more common Pearson correlation. We find that activating a strategy is moderately-to-strongly correlated with a suppression in the corresponding error mechanism ($-0.67^{+0.02}_{-0.02} \leq C_{m,s} \leq -0.59^{+0.03}_{-0.03}$ for diagonals) while being only weakly correlated with other mechanisms ($-0.07^{+0.04}_{-0.04} \leq C_{m,s} \leq 0.15^{+0.04}_{-0.04}$ for off-diagonals).

Our second metric is the relative error contribution $\Delta_{m,s} = e_{m,s=1}/e_{m,s=0} - 1$ (Supplementary Fig. 16c). Here $e_{m,s=1(0)} = \langle e_{m,ij} \rangle_{ij,s=1(0)}$ is the median over pairs in all configurations where strategy s is active (inactive). We find that activating a particular strategy moderately-to-strongly suppresses its corresponding error mechanism ($-0.75^{+0.07}_{-0.07} \leq \Delta_{m,s} \leq -0.33^{+0.03}_{-0.03}$ for diagonals), while relatively weakly interacting with others ($-0.15^{+0.12}_{-0.13} \leq \Delta_{m,s} \leq 0.26^{+0.11}_{-0.11}$ for off-diagonals). Interestingly, relaxation mitigation appears to be less effective than other mitigation strategies ($\Delta_{m,s} = -0.33^{+0.03}_{-0.03}$ with $m = s =$relaxation). However, the last panel of Supplementary Fig. 15b actually shows that relaxation approaches its suppression limit, even when all mitigation strategies are active, suggesting that relaxation is limited by our processor's performance limits and not our optimizer.

These results suggest that our mitigation strategies are both selective and effective at suppressing their corresponding error mechanisms. In turn, they support our association of error components with mitigation strategies and that our optimizer can effectively navigate them.

8.5. Scaling experiment

When building configurations for the scaling experiment in Fig. 4, multiple configurations were used for smaller configuration sizes ($N < 40$) to boost statistics (configurations column in Supplementary Table 3). Optimized configurations were generally healed one or more times to resolve calibration failures and to push the performance limits of our processor. However, frequencies were never selected manually. Unoptimized baseline configurations were never healed, leading $\sim 1\%$ of all gates across all baseline configurations to fail calibrations.

The best-fit parameters for the saturation model are in Supplementary Table 5. The parameters e_{sat} and e_{scale} are remarkably similar between experiment and simulation. However, there is a sizeable gap for N_{sat}. We trust the experimental value more since the simulated data are sparse towards smaller N (i.e. the $d = 3, 5$, and 7 logical qubits have $N = 7, 49$, and 96, respectively).

8.6. Stitching experiment

The stitching demonstrations in Fig. 4 employed convenient stitch geometries. Even though stitched-and-healed configurations performed as well as their unstitched counterparts, we expect that the number of stitched regions and seam geometry will ultimately need to be optimized. First, we expect that progressively increasing the number of stitched regions - which would favorably lead to shorter optimization runtimes - will eventually start to degrade performance as more constraints between more independently optimized configurations will have to be reconciled. Second, we expect that optimizing the seam geometry will be necessary for applications like error correction, where the geometry of underperformant gates is particularly important[55,60].

8.7. Algorithm specificity

Ideally a single frequency configuration could reach high performance on arbitrary quantum algorithms. However, that configuration would have to mitigate frequency collisions between all possible combinations of parasitically coupled gates. That stringent requirement may be infeasible from a constraint perspective.

To test this possibility, we optimize our processor for arbitrary quantum algorithms by configuring the stray coupling error components to penalize for frequency collisions between all possible combinations of parasitically coupled SQ and CZ gates. We compare the performance of the configuration against configurations optimized without stray coupling mitigation, with the default CZXEB-specific stray coupling mitigation, and the baseline, outlier, and crossover standards.

The configuration optimized for arbitrary quantum algorithms significantly underperforms the default configuration optimized with CZXEB-specific stray coupling mitigation and the crossover standard. Furthermore, it only moderately outperforms the configuration without stray coupling mitigation at all and the random baseline standard (Supplementary Fig. 17). This result suggests that the added stray coupling error components overconstrain the optimization problem. Therefore, with the current magnitude of stray coupling, algorithm-specific optimization is critical.

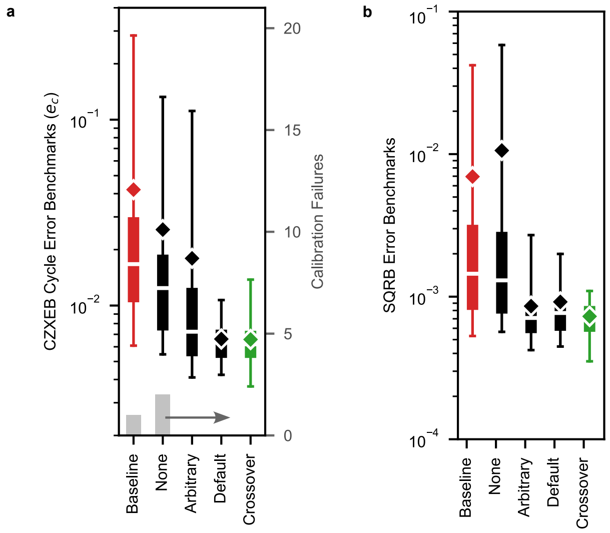

Supplementary Fig. 17. Impact of algorithm-specific optimization. (a) CZXEB cycle error and (b) SQRB error benchmarks for configurations optimized without stray coupling mitigation (None), with arbitrary-algorithm stray coupling mitigation (Arbitrary), and the default CZXEB-specific stray coupling mitigation (Default). The baseline (red), outlier (orange), and crossover standards are shown for comparison. Calibration failures are grey bars (right axis).

8.8. Dependencies

Optimization was performed on an Lenovo ThinkStation P620 with 128GB of ram and an AMD Ryzen Threadripper PRO 3945WX. Data were analyzed using a combination of `numpy`[61], `scipy`[62], `pandas`[63], and `matplotlib`[64]. Boxplots were generated using `matplotlib.boxplot`. The percentiles in Supplementary Tables 1-4 were generated using `numpy.percentile`. The tested inner-loop optimizers leveraged a mixture of homebrew code and `scipy`'s built-in optimizers. The training pipeline was written in `tensorflow`[37].

Supplementary Table 1. **Optimization and healing performance**. CZXEB cycle error and SQRB error benchmarks ($\times 10^{-3}$) corresponding to the configurations in Fig. 2.

Benchmark	N	Label	Scope (S)	Min	Max	Mean	2.5%	25%	50%	75%	97.5%
CZXEB	68	Baseline	-	3.7	300.0	42.0	6.1	10.7	16.7	29.4	283.8
CZXEB	-	Outlier	-	-	-	15.0	-	-	-	-	-
CZXEB	49	Crossover	-	2.5	14.8	6.6	3.7	5.3	6.2	7.2	13.8
CZXEB	68	Optimized	1	2.5	300.0	25.2	3.7	7.4	9.8	15.5	241.6
CZXEB	68	Optimized	2	4.0	64.3	9.2	4.7	5.7	7.2	9.3	27.0
CZXEB	68	Optimized	3	3.7	29.4	9.0	4.8	6.3	7.4	9.8	20.9
CZXEB	68	Optimized	4	3.9	31.4	8.5	4.1	5.5	6.8	9.5	26.1
CZXEB	68	Optimized	5	2.5	295.0	20.4	3.9	6.3	7.6	12.2	136.4
CZXEB	68	Optimized	6	2.5	181.5	20.2	4.2	6.4	8.4	14.8	122.7
CZXEB	68	Optimized	S_{max}	3.5	299.9	27.1	5.2	8.6	10.8	19.2	155.8
CZXEB	68	Healed	1	4.6	49.6	10.6	5.0	7.5	8.7	11.6	23.5
CZXEB	68	Healed	2	3.9	54.6	8.6	4.4	6.0	7.2	9.0	20.5
CZXEB	68	Healed	3	3.7	91.2	9.2	4.4	6.2	7.7	9.1	22.6
CZXEB	68	Healed	4	2.7	62.8	8.5	4.0	5.6	6.9	8.6	18.8
CZXEB	68	Healed	5	2.5	53.6	8.7	4.3	5.6	7.3	9.3	18.9
CZXEB	68	Healed	6	2.5	275.2	12.7	3.8	5.6	6.8	9.9	31.3
CZXEB	68	Healed	S_{max}	3.5	124.4	14.0	4.5	6.8	8.8	13.1	66.7
SQRB	49	Baseline	-	0.43	53.19	6.96	0.53	0.83	1.45	3.07	42.02
SQRB	-	Outlier	-	-	-	-	-	-	-	-	-
SQRB	49	Crossover	-	0.33	1.47	0.73	0.35	0.58	0.72	0.84	1.10
SQRB	68	Optimized	1	0.40	12.75	1.26	0.42	0.61	0.73	1.04	3.86
SQRB	68	Optimized	2	0.26	25.74	1.24	0.40	0.58	0.68	0.87	2.67
SQRB	68	Optimized	3	0.36	13.87	1.21	0.44	0.58	0.70	0.82	3.99
SQRB	68	Optimized	4	0.39	27.49	1.41	0.48	0.62	0.76	0.94	3.41
SQRB	68	Optimized	5	0.36	4.61	1.00	0.38	0.61	0.72	1.03	3.22
SQRB	68	Optimized	6	0.00	11.14	1.20	0.34	0.56	0.67	0.94	3.82
SQRB	68	Optimized	S_{max}	0.38	91.07	3.90	0.53	0.80	1.12	1.65	21.30
SQRB	68	Healed	1	0.38	7.80	1.09	0.47	0.64	0.72	1.06	3.62
SQRB	68	Healed	2	0.34	4.11	0.86	0.40	0.56	0.74	0.86	1.94
SQRB	68	Healed	3	0.40	9.92	0.90	0.43	0.58	0.68	0.80	1.60
SQRB	68	Healed	4	0.32	2.82	0.88	0.41	0.61	0.72	0.96	2.51
SQRB	68	Healed	5	0.49	4.10	1.08	0.53	0.70	0.97	1.18	2.73
SQRB	68	Healed	6	0.04	1.93	0.78	0.49	0.61	0.75	0.85	1.36
SQRB	68	Healed	S_{max}	0.29	4.72	0.86	0.40	0.61	0.73	0.90	1.66

Supplementary Table 2. **Optimization performance for various error mitigation strategies**. CZXEB cycle error and SQRB error benchmarks ($\times 10^{-3}$) corresponding to the configurations in Fig. 3.

Benchmark	N	Dephasing	Relaxation	Stray coupling	Pulse distortion	Min	Max	Mean	2.5%	25%	50%	75%	97.5%
CZXEB	-	Outlier				-	-	15.0	-	-	-	-	-
CZXEB	68	Baseline				3.7	300.0	42.0	6.1	10.7	16.7	29.4	283.8
CZXEB	68	1	0	0	0	2.5	299.9	42.2	2.7	7.5	21.5	43.1	222.2
CZXEB	68	0	1	0	0	2.5	285.1	20.7	3.7	7.4	9.3	18.2	92.7
CZXEB	68	0	0	1	0	2.5	283.2	29.9	3.9	8.2	14.8	29.4	192.1
CZXEB	68	0	0	0	1	2.5	299.8	53.2	2.5	3.8	15.4	54.7	266.2
CZXEB	68	1	1	0	0	2.5	277.6	32.1	5.2	8.2	13.4	29.4	184.1
CZXEB	68	1	0	1	0	3.7	287.4	19.1	4.1	7.2	9.2	12.8	73.8
CZXEB	68	1	0	0	1	3.7	237.3	28.5	3.7	8.0	14.8	30.1	127.7
CZXEB	68	0	1	1	0	2.5	286.7	14.9	4.2	5.9	8.0	14.2	46.9
CZXEB	68	0	1	0	1	2.5	299.7	25.7	3.7	7.4	12.6	18.8	124.9
CZXEB	68	0	0	1	1	2.5	139.9	12.1	4.0	5.2	6.3	9.2	52.7
CZXEB	68	1	1	1	0	3.7	27.3	8.1	4.2	5.8	7.4	9.3	16.5
CZXEB	68	1	1	0	1	3.7	299.0	25.6	5.5	7.5	12.4	18.0	132.6
CZXEB	68	1	0	1	1	3.6	224.0	15.1	3.7	6.0	8.2	12.4	79.0
CZXEB	68	0	1	1	1	3.8	102.4	8.2	4.2	5.2	6.2	7.3	24.8
CZXEB	68	1	1	1	1	4.0	14.5	6.6	4.2	5.3	6.4	7.3	10.7
CZXEB	49	Crossover				2.5	14.8	6.6	3.7	5.3	6.2	7.2	13.8
SQRB	-	Outlier				-	-	-	-	-	-	-	-
SQRB	68	Baseline				0.43	53.19	6.96	0.53	0.83	1.45	3.07	42.02
SQRB	68	1	0	0	0	0.05	74.55	12.99	0.59	1.70	6.86	20.22	53.15
SQRB	68	0	1	0	0	0.53	38.31	2.19	0.55	0.72	0.84	1.17	9.16
SQRB	68	0	0	1	0	0.01	46.63	5.37	0.44	0.67	0.99	3.02	35.67
SQRB	68	0	0	0	1	0.08	188.21	33.32	1.30	12.50	26.52	50.78	76.57
SQRB	68	1	1	0	0	0.45	44.92	5.17	0.48	0.79	1.79	3.39	34.88
SQRB	68	1	0	1	0	0.40	61.34	2.72	0.45	0.59	0.73	1.12	21.52
SQRB	68	1	0	0	1	0.47	65.69	5.51	0.52	0.86	1.50	5.33	19.94
SQRB	68	0	1	1	0	0.02	38.28	3.30	0.40	0.61	0.74	1.01	27.70
SQRB	68	0	1	0	1	0.47	51.42	3.67	0.52	0.78	1.17	2.15	20.09
SQRB	68	0	0	1	1	0.29	61.05	1.96	0.47	0.61	0.73	0.89	4.59
SQRB	68	1	1	1	0	0.41	3.95	0.95	0.43	0.63	0.76	1.12	2.43
SQRB	68	1	1	0	1	0.47	199.96	10.60	0.57	0.78	1.30	2.79	58.24
SQRB	68	1	0	1	1	0.01	33.36	2.20	0.46	0.61	0.73	1.08	9.42
SQRB	68	0	1	1	1	0.29	7.45	0.87	0.42	0.54	0.69	0.82	2.28
SQRB	68	1	1	1	1	0.34	4.64	0.92	0.45	0.59	0.77	0.96	1.99
SQRB	49	Crossover				0.33	1.47	0.73	0.35	0.58	0.72	0.84	1.10

Supplementary Table 3. **Optimization scalability experiment**. CZXEB cycle error and SQRB error benchmarks ($\times 10^{-3}$) corresponding to the configurations in Fig. 4a.

Benchmark	N	Configurations	Label	Min	Max	Mean	2.5%	25%	50%	75%	97.5%
CZXEB	-	-	Outlier	-	-	15.0	-	-	-	-	-
CZXEB	2	94	Optimized	2.7	23.6	5.0	3.0	3.7	4.3	5.4	10.2
CZXEB	4	39	Optimized	2.8	17.7	4.9	2.9	3.8	4.5	5.3	9.0
CZXEB	9	15	Optimized	2.5	11.6	5.0	3.4	3.9	4.5	5.6	8.5
CZXEB	16	5	Optimized	3.2	15.6	5.9	3.5	4.7	5.4	6.8	10.2
CZXEB	24	6	Optimized	3.1	31.9	6.5	3.6	4.5	5.8	7.2	14.9
CZXEB	33	2	Optimized	3.2	16.7	6.7	3.9	4.8	6.0	7.5	14.4
CZXEB	40	1	Optimized	3.9	16.5	7.4	4.3	5.6	6.8	8.9	12.9
CZXEB	47	1	Optimized	3.7	15.2	7.5	4.3	5.6	6.8	8.7	14.8
CZXEB	49	-	Crossover	2.5	14.8	6.6	3.7	5.3	6.2	7.2	13.8
CZXEB	54	1	Optimized	3.7	29.4	7.4	4.4	5.8	6.9	8.1	13.7
CZXEB	62	1	Optimized	3.7	17.9	7.6	4.7	6.0	7.2	8.4	12.8
CZXEB	68	1	Optimized	4.0	14.5	6.6	4.2	5.3	6.4	7.3	10.7
CZXEB	68	1	Stitched ($R = 2$)	4.2	20.6	7.0	4.6	5.5	6.4	7.7	10.8
CZXEB	2	109	Baseline	2.2	157.1	15.5	3.4	4.6	5.8	10.2	95.5
CZXEB	4	37	Baseline	3.4	97.6	9.5	3.9	4.9	6.8	9.1	34.4
CZXEB	9	18	Baseline	3.0	300.0	15.2	4.2	6.2	7.8	12.7	85.7
CZXEB	16	6	Baseline	2.5	291.4	25.1	4.7	7.5	10.8	18.1	135.9
CZXEB	24	7	Baseline	3.1	299.1	20.2	5.1	8.1	12.5	18.2	82.1
CZXEB	33	3	Baseline	2.5	295.7	26.9	3.7	7.9	14.0	28.2	124.0
CZXEB	44	1	Baseline	2.5	184.0	24.2	3.7	8.3	13.9	25.4	126.6
CZXEB	47	1	Baseline	4.0	293.4	24.4	5.3	9.4	14.8	26.7	75.6
CZXEB	54	1	Baseline	2.5	288.9	21.0	3.7	7.6	11.6	14.8	102.4
CZXEB	62	1	Baseline	2.5	277.8	33.4	6.7	13.0	16.8	29.4	221.4
CZXEB	68	1	Baseline	3.7	300.0	42.0	6.1	10.7	16.7	29.4	283.8
SQRB	-	-	Outlier	-	-	-	-	-	-	-	-
SQRB	2	94	Optimized	0.29	6.43	0.72	0.37	0.51	0.60	0.71	1.72
SQRB	4	39	Optimized	0.35	3.07	0.65	0.41	0.52	0.61	0.72	1.03
SQRB	9	15	Optimized	0.31	2.48	0.69	0.42	0.54	0.62	0.72	1.72
SQRB	16	5	Optimized	0.36	2.02	0.77	0.44	0.55	0.67	0.85	1.69
SQRB	24	6	Optimized	0.32	7.14	0.81	0.42	0.59	0.69	0.86	1.58
SQRB	33	2	Optimized	0.24	2.84	0.87	0.34	0.63	0.73	0.84	2.38
SQRB	40	1	Optimized	0.41	2.10	0.82	0.46	0.58	0.70	0.89	1.94
SQRB	47	1	Optimized	0.22	1.44	0.73	0.36	0.60	0.71	0.88	1.12
SQRB	49	-	Crossover	0.33	1.47	0.73	0.35	0.58	0.72	0.84	1.10
SQRB	54	1	Optimized	0.31	1.62	0.77	0.48	0.58	0.71	0.85	1.30
SQRB	62	1	Optimized	0.37	2.74	0.88	0.48	0.64	0.80	1.00	1.59
SQRB	68	1	Optimized	0.34	4.64	0.92	0.45	0.59	0.77	0.96	1.99
SQRB	68	1	Stitched ($R = 2$)	0.41	3.23	0.88	0.44	0.61	0.75	0.93	2.16
SQRB	2	109	Baseline	0.28	21.74	1.04	0.37	0.56	0.65	0.85	3.36
SQRB	4	37	Baseline	0.28	5.87	0.99	0.41	0.59	0.75	1.14	2.19
SQRB	9	18	Baseline	0.32	50.40	2.12	0.46	0.65	0.79	1.41	12.15
SQRB	16	6	Baseline	0.37	60.85	3.85	0.50	0.75	1.08	2.06	23.35
SQRB	24	7	Baseline	0.30	53.72	2.80	0.45	0.70	1.02	1.85	16.22
SQRB	33	3	Baseline	0.43	46.46	3.66	0.56	0.77	1.10	2.14	21.29
SQRB	44	1	Baseline	0.01	16.37	2.37	0.49	0.72	0.90	1.55	10.36
SQRB	47	1	Baseline	0.00	18.90	2.49	0.52	0.81	1.10	2.17	12.68
SQRB	54	1	Baseline	0.30	25.93	2.06	0.50	0.72	1.24	1.74	10.16
SQRB	62	1	Baseline	0.48	76.72	5.93	0.53	0.96	1.61	4.40	25.50
SQRB	68	1	Baseline	0.43	53.19	6.96	0.53	0.83	1.45	3.07	42.02

Supplementary Table 4. **Optimization scalability simulation**. CZXEB cycle error and SQRB error benchmarks ($\times 10^{-3}$) corresponding to the configurations in Fig. 4b.

Benchmark	N	Distance	Configurations	Label	Min	Max	Mean	2.5%	25%	50%	75%	97.5%
CZXEB	-	-	-	Outlier	-	-	15.0	-	-	-	-	-
CZXEB	17	3	1	Optimized	3.3	10.0	5.5	3.8	4.6	5.6	6.0	7.2
CZXEB	49	5	1	Optimized	3.4	12.6	6.3	3.6	5.1	6.1	7.3	10.6
CZXEB	49	5	1	Crossover	2.5	14.8	6.6	3.7	5.3	6.2	7.2	13.8
CZXEB	97	7	1	Optimized	3.1	25.3	7.2	3.8	5.4	6.9	8.3	11.9
CZXEB	161	9	1	Optimized	3.3	13.6	6.6	3.8	5.4	6.6	7.6	10.7
CZXEB	241	11	1	Optimized	2.9	15.7	7.2	4.0	5.7	6.9	8.3	11.9
CZXEB	337	13	1	Optimized	3.0	74.6	7.6	3.8	5.6	6.9	8.3	11.5
CZXEB	449	15	1	Optimized	2.7	191.0	8.2	3.8	5.8	6.9	8.3	12.1
CZXEB	577	17	1	Optimized	3.0	53.5	7.4	4.0	5.9	7.2	8.4	11.2
CZXEB	721	19	1	Optimized	3.0	33.8	7.4	4.1	6.0	7.1	8.4	11.9
CZXEB	881	21	1	Optimized	2.7	66.3	7.3	4.0	5.8	7.0	8.2	11.2
CZXEB	1057	23	1	Optimized	2.9	50.9	7.4	4.2	6.0	7.2	8.4	11.8
CZXEB	1057	23	1	Stitched ($R = 4$)	2.6	50.4	6.5	3.4	5.2	6.3	7.5	10.6
CZXEB	17	3	1	Baseline	3.4	44.1	16.0	5.5	9.1	11.6	17.4	40.3
CZXEB	49	5	1	Baseline	4.1	87.3	19.3	6.7	10.5	15.7	21.1	61.6
CZXEB	97	7	1	Baseline	3.7	135.7	23.0	6.0	10.4	13.8	23.0	108.4
CZXEB	161	9	1	Baseline	4.0	252.1	22.0	5.9	11.0	14.3	21.8	89.9
CZXEB	241	11	1	Baseline	5.7	934.7	30.6	7.3	11.6	15.5	22.7	108.6
CZXEB	337	13	1	Baseline	3.6	341.3	21.5	6.6	11.4	14.9	21.0	78.7
CZXEB	449	15	1	Baseline	4.7	348.0	23.6	6.8	11.1	14.4	19.9	113.9
CZXEB	577	17	1	Baseline	3.8	448.0	22.9	6.5	10.9	14.6	20.7	101.4
CZXEB	721	19	1	Baseline	3.8	381.3	23.6	6.3	11.1	14.5	20.7	109.9
CZXEB	881	21	1	Baseline	4.2	543.9	26.0	6.3	11.1	14.8	21.0	127.3
CZXEB	1057	23	1	Baseline	3.7	888.3	23.6	6.8	11.3	14.8	20.4	115.4
SQRB	-	-	-	Outlier	-	-	-	-	-	-	-	-
SQRB	17	3	1	Optimized	0.53	0.96	0.72	0.56	0.63	0.71	0.83	0.89
SQRB	49	5	1	Optimized	0.53	2.11	0.78	0.54	0.63	0.73	0.86	1.10
SQRB	49	5	-	Crossover	0.33	1.47	0.73	0.35	0.58	0.72	0.84	1.10
SQRB	97	7	1	Optimized	0.52	13.11	0.89	0.53	0.65	0.73	0.83	1.26
SQRB	161	9	1	Optimized	0.51	2.44	0.81	0.54	0.69	0.78	0.88	1.38
SQRB	241	11	1	Optimized	0.49	1.61	0.78	0.56	0.69	0.76	0.85	1.10
SQRB	337	13	1	Optimized	0.49	44.00	0.98	0.55	0.70	0.78	0.88	1.30
SQRB	449	15	1	Optimized	0.43	74.91	1.00	0.55	0.69	0.77	0.88	1.29
SQRB	577	17	1	Optimized	0.44	4.28	0.81	0.56	0.69	0.77	0.87	1.24
SQRB	721	19	1	Optimized	0.47	6.52	0.84	0.56	0.70	0.80	0.90	1.24
SQRB	881	21	1	Optimized	0.44	35.55	0.84	0.56	0.70	0.79	0.87	1.19
SQRB	1057	23	1	Optimized	0.45	5.58	0.83	0.56	0.70	0.79	0.90	1.23
SQRB	1057	23	1	Stitched ($R = 4$)	0.43	5.58	0.82	0.57	0.71	0.79	0.88	1.16
SQRB	17	3	1	Baseline	0.53	10.42	1.71	0.62	0.89	1.23	1.38	2.37
SQRB	49	5	1	Baseline	0.63	13.47	1.61	0.72	0.87	1.03	1.41	5.85
SQRB	97	7	1	Baseline	0.52	78.62	2.80	0.57	0.91	1.11	1.44	10.47
SQRB	161	9	1	Baseline	0.51	78.78	2.45	0.61	0.89	1.08	1.43	6.94
SQRB	241	11	1	Baseline	0.52	879.29	6.06	0.61	0.92	1.21	1.61	15.20
SQRB	337	13	1	Baseline	0.46	80.89	2.41	0.58	0.95	1.28	1.67	8.21
SQRB	449	15	1	Baseline	0.46	80.73	2.34	0.63	0.91	1.26	1.69	10.71
SQRB	577	17	1	Baseline	0.44	119.90	2.63	0.60	0.91	1.17	1.56	9.10
SQRB	721	19	1	Baseline	0.48	348.00	3.10	0.63	0.93	1.22	1.68	21.58
SQRB	881	21	1	Baseline	0.49	393.14	4.01	0.62	0.96	1.24	1.70	15.74
SQRB	1057	23	1	Baseline	0.45	199.55	3.02	0.62	0.93	1.21	1.62	13.30

Supplementary Table 5. **Scaling model parameters**. Best-fit parameters for the saturation model introduced in the manuscript and plotted in Fig. 4a and 4b. The error bars are standard deviations ($\pm 1\sigma$).

	Experiment		Simulation	
	Baseline	Optimized	Baseline	Optimized
N_{sat}	21 ± 21	22 ± 10	49 ± 46	84 ± 41
$e_{\text{sat}} \times 10^{-3}$	28 ± 5	7.5 ± 0.4	24 ± 1	7.5 ± 0.2
$e_{\text{scale}} \times 10^{-3}$	17 ± 5	3.1 ± 0.4	12 ± 6	2.2 ± 0.6

Supplementary Table 6. **Symbol definitions**. Definitions for symbols used in the manuscript and Supplementary Information.

Symbol	Definition
S	Snake's scope parameter
q_i	Qubit with index i
SQ_i	Single-qubit gate executed by q_i
CZ_{ij}	Controlled-Z gate executed by q_i and q_j
N	Number of qubits in a configuration
d	Distance of a surface-code logical qubit
f_i	Idle frequency for q_i
f_{ij}	Interaction frequency for q_i and q_j
F	Set of f_i and f_{ij} corresponding to a frequency configuration
F^*	Set of optimized gate frequencies corresponding to F
F_g	Subset of F that are relevant to estimating the error of gate g
$F_{g,m}$	Subset of F_g that are relevant to estimating the error of gate for physical error mechanism m
F_S	Subset of F selected for optimization by Snake based on scope S
k	Approximate number of frequency options per gate
e_i	Error per gate for SQ_i, measured by SQRB and reported as an average error
e_{ij}	Error per gate for CZ_{ij}, which can be inferred from SQRB and CZXEB benchmarks
$e_{c,ij}$	Error per cycle for SQ_i, SQ_j, and CZ_{ij}, measured by CZXEB and reported as an average error
e_c	Distribution of $e_{c,ij}$ corresponding to some configuration(s)
N_{sat}	Qubit saturation constant in the heuristic saturation scaling model
e_{sat}	Saturated error in the heuristic saturation scaling model
e_{scale}	Scaling penalty in the heuristic saturation scaling model
D	Characterization data taken by the control system
T_1	Energy-relaxation time
T_ϕ	Dephasing time as inferred from the flux sensitivity $\frac{df}{d\phi}$
χ	Stray coupling parameters between parasitically coupled gates
δ	Frequency-pulse distortion parameters
$\mathcal{F}$	Frequency trajectory used to implement a CZ gate
A	Quantum circuit(s) for the target quantum algorithm(s)
g	Some gate in A
E	Algorithm error estimator for A
E_g	Gate error estimator for gate g
E_S	Snake estimator that generally comprises some terms in E
M	Set of physical error mechanisms m
m	One physical error mechanism
$\epsilon_{g,m}$	Algorithm-independent error component for gate g and physical error mechanism m
$w_{g,m}$	Algorithm-dependent weight corresponding to $\epsilon_{g,m}$
P	Probability density, used in the context of simulated processors
$\mathcal{N}$	Processor size and connectivity, used in the context of simulated processors
$\mathcal{P}$	Processor architectural parameters, used in the context of simulated processors
s	Error mitigation strategy
$e_{m,ij}$	Error per cycle for SQ_i, SQ_j, and CZ_{ij} for error mechanism m, estimated from the error components
e_m	Median of $e_{m,ij}$ taken over pairs ij
$\Delta_{m,s}$	Relative error contribution from mechanism m when strategy s is active
$C_{m,s}$	Correlation between the activation of error mitigation strategy s and error mechanism m
$\text{argmin}_x(f(x))$	Minimizer for some function $f(x)$ with respect to its argument(s) x
$\lvert . \rvert$	Cardinality of the set .
$\langle . \rangle$	Expectation value of .
σ	Standard deviation

1. Arute, F. *et al.* Quantum supremacy using a programmable superconducting processor. *Nature* **574**, 505–510 (2019).
2. Acharya, R. *et al.* Suppressing quantum errors by scaling a surface code logical qubit. *Nature* **614**, 676–681 (2023).
3. Kelly, J., O'Malley, P., Neeley, M., Neven, H. & Martinis, J. M. Physical qubit calibration on a directed acyclic graph (2018). Preprint at http://arXiv.org/abs/1803.03226.
4. Foxen, B. *et al.* Demonstrating a continuous set of two-qubit gates for near-term quantum algorithms. *Phys. Rev. Lett.* **125**, 120504 (2020).
5. Boixo, S. *et al.* Characterizing quantum supremacy in near-term devices. *Nature Physics* **14**, 595–600 (2018).
6. Hastie, T., Tibshirani, R. & Friedman, J. *The Elements of Statistical Learning: Data Mining, Inference, and Prediction, Second Edition.* Springer Series in Statistics (Springer New York, 2009).
7. Russell, ., Stuart J. (Stuart Jonathan). *Artificial intelligence : a modern approach* (Third edition. Upper Saddle River, N.J. : Prentice Hall, [2010] ©2010, [2010]).
8. James, G., Witten, D., Hastie, T. & Tibshirani, R. *An Introduction to Statistical Learning: with Applications in R* (Springer, 2013).
9. Wallman, J. J. & Emerson, J. Noise tailoring for scalable quantum computation via randomized compiling. *Phys. Rev. A* **94**, 052325 (2016).
10. Hashim, A. *et al.* Randomized compiling for scalable quantum computing on a noisy superconducting quantum processor. *Phys. Rev. X* **11**, 041039 (2021).
11. Jain, A., Iyer, P., Bartlett, S. D. & Emerson, J. Improved quantum error correction with randomized compiling. *Phys. Rev. Res.* **5**, 033049 (2023).
12. O'Malley, P. J. J. *et al.* Qubit metrology of ultralow phase noise using randomized benchmarking. *Phys. Rev. Appl.* **3**, 044009 (2015).
13. Ithier, G. *et al.* Decoherence in a superconducting quantum bit circuit. *Phys. Rev. B* **72**, 134519 (2005).
14. Bylander, J. *et al.* Noise spectroscopy through dynamical decoupling with a superconducting flux qubit. *Nature Physics* **7**, 565–570 (2011).
15. Müller, C., Cole, J. H. & Lisenfeld, J. Towards understanding two-level-systems in amorphous solids: insights from quantum circuits. *Reports on Progress in Physics* **82**, 124501 (2019).
16. Niu, M. Y. *et al.* Learning non-markovian quantum noise from moiré-enhanced swap spectroscopy with deep evolutionary algorithm (2019). Preprint at http://arXiv.org/abs/1912.04368.
17. Abad, T., Fernández-Pendás, J., Frisk Kockum, A. & Johansson, G. Universal fidelity reduction of quantum operations from weak dissipation. *Phys. Rev. Lett.* **129**, 150504 (2022).
18. Marxer, F. *et al.* Long-distance transmon coupler with cz-gate fidelity above 99.8%. *PRX Quantum* **4**, 010314 (2023).
19. Mundada, P., Zhang, G., Hazard, T. & Houck, A. Suppression of qubit crosstalk in a tunable coupling superconducting circuit. *Phys. Rev. Appl.* **12**, 054023 (2019).
20. Rol, M. A. *et al.* Time-domain characterization and correction of on-chip distortion of control pulses in a quantum processor. *Applied Physics Letters* **116**, 054001 (2020).
21. Foxen, B. *et al.* High speed flux sampling for tunable superconducting qubits with an embedded cryogenic transducer. *Superconductor Science and Technology* **32**, 015012 (2018).
22. Koch, J. *et al.* Charge-insensitive qubit design derived from the cooper pair box. *Phys. Rev. A* **76**, 042319 (2007).
23. Krantz, P. *et al.* A quantum engineer's guide to superconducting qubits. *Applied Physics Reviews* **6**, 021318 (2019).
24. Bluvstein, D. *et al.* A quantum processor based on coherent transport of entangled atom arrays. *Nature* **604**, 451–456 (2022).
25. Bluvstein, D. *et al.* Logical quantum processor based on reconfigurable atom arrays. *Nature* (2023).
26. Mills, A. R. *et al.* Shuttling a single charge across a one-dimensional array of silicon quantum dots. *Nature Communications* **10**, 1063 (2019).
27. Zwolak, J. P. & Taylor, J. M. Colloquium: Advances in automation of quantum dot devices control. *Rev. Mod. Phys.* **95**, 011006 (2023).
28. Zwanenburg, F. A. *et al.* Silicon quantum electronics. *Reviews of Modern Physics* **85**, 961–1019 (2013).
29. Burkard, G., Ladd, T. D., Pan, A., Nichol, J. M. & Petta, J. R. Semiconductor spin qubits. *Reviews of Modern Physics* **95** (2023).
30. Durandau, J. *et al.* Automated Generation of Shuttling Sequences for a Linear Segmented Ion Trap Quantum Computer. *Quantum* **7**, 1175 (2023).
31. Kreppel, F. *et al.* Quantum circuit compiler for a shuttling-based trapped-ion quantum computer. *Quantum* **7**, 1176 (2023).
32. Sterk, J. D. *et al.* Closed-loop optimization of fast trapped-ion shuttling with sub-quanta excitation. *npj Quantum Information* **8**, 68 (2022).
33. Qi, L. *Optimizing Ion-shuttling Operations in Trapped-ion Quantum Computers.* Thesis, Massachusetts Institute of Technology (2021).
34. Mohri, M., Rostamizadeh, A. & Talwalkar, A. *Foundations of Machine Learning.* Adaptive Computation and Machine Learning (MIT Press, Cambridge, MA, 2018), 2 edn.
35. Klimov, P. V., Megrant, A. E., Dunsworth, A. L. & Kelly, J. S. Generative modeling of quantum hardware. *US Patent* US20230259802A1 (2020).
36. Klimov, P. V. Iterative supervised learning of quantum processor error models. *US Patent* US20230359922A1 (2022).
37. Abadi, M. *et al.* TensorFlow: Large-scale machine learning on heterogeneous systems (2015). URL `https://www.tensorflow.org/`. Software available from tensorflow.org.
38. Kingma, D. P. & Ba, J. Adam: A method for stochastic optimization (2017). Preprint at http://arXiv.org/abs/1412.6980.
39. Klimov, P. V. *et al.* Fluctuations of energy-relaxation times in superconducting qubits. *Phys. Rev. Lett.* **121**, 090502 (2018).
40. Klimov, P. V. Calibration of quantum processor operator parameters. *US Patent* US20200387822A1 (2019).

41. Klimov, P. V., Kelly, J., Martinis, J. M. & Neven, H. The snake optimizer for learning quantum processor control parameters (2020). Preprint at http://arXiv.org/abs/2006.04594.
42. Jeffrey, E. *et al.* Fast accurate state measurement with superconducting qubits. *Phys. Rev. Lett.* **112**, 190504 (2014).
43. Cormen, T. H., Leiserson, C. E., Rivest, R. L. & Stein, C. *Introduction to Algorithms* (The MIT Press, 2001), 2 edn.
44. Sutton, R. S. & Barto, A. G. *Reinforcement Learning: An Introduction* (The MIT Press, 2018), second edn.
45. Storn, R. & Price, K. Differential evolution – a simple and efficient heuristic for global optimization over continuous spaces. *Journal of Global Optimization* **11**, 341–359 (1997).
46. Liu, D. C. & Nocedal, J. On the limited memory bfgs method for large scale optimization. *Mathematical Programming* **45**, 503–528 (1989).
47. Endres, S., Sandrock, C. & Focke, W. A simplicial homology algorithm for lipschitz optimisation. *Journal of Global Optimization* **72** (2018).
48. Tsallis, C. & Stariolo, D. A. Generalized simulated annealing. *Physica A: Statistical Mechanics and its Applications* **233**, 395–406 (1996).
49. Jones, D. R., Perttunen, C. D. & Stuckman, B. E. Lipschitzian optimization without the lipschitz constant. *Journal of Optimization Theory and Applications* **79**, 157–181 (1993).
50. Wales, D. J. & Doye, J. P. K. Global optimization by basin-hopping and the lowest energy structures of lennard-jones clusters containing up to 110 atoms. *The Journal of Physical Chemistry A* **101**, 5111–5116 (1997).
51. Sivak, V. V. *et al.* Model-free quantum control with reinforcement learning. *Phys. Rev. X* **12**, 011059 (2022).
52. Baum, Y. *et al.* Experimental deep reinforcement learning for error-robust gate-set design on a superconducting quantum computer. *PRX Quantum* **2** (2021).
53. Ball, H. *et al.* Software tools for quantum control: improving quantum computer performance through noise and error suppression. *Quantum Science and Technology* **6**, 044011 (2021).
54. Hothem, D., Hines, J., Nataraj, K., Blume-Kohout, R. & Proctor, T. Predictive models from quantum computer benchmarks. In *2023 IEEE International Conference on Quantum Computing and Engineering (QCE)*, 709–714 (IEEE Computer Society, Los Alamitos, CA, USA, 2023).
55. Nagayama, S., Fowler, A. G., Horsman, D., Devitt, S. J. & Meter, R. V. Surface code error correction on a defective lattice. *New Journal of Physics* **19**, 023050 (2017).
56. Emerson, J., Alicki, R. & Życzkowski, K. Scalable noise estimation with random unitary operators. *Journal of Optics B: Quantum and Semiclassical Optics* **7**, S347 (2005).
57. DiCarlo, L. *et al.* Demonstration of two-qubit algorithms with a superconducting quantum processor. *Nature* **460**, 240–244 (2009).
58. Purcell, E. M. Spontaneous Emission Probabilities at Radio Frequencies. *Physical Review* **69**, 681 (1946).
59. Huang, S. *et al.* Microwave package design for superconducting quantum processors. *PRX Quantum* **2**, 020306 (2021).
60. Fowler, A. G., Mariantoni, M., Martinis, J. M. & Cleland, A. N. Surface codes: Towards practical large-scale quantum computation. *Phys. Rev. A* **86**, 032324 (2012).
61. Harris, C. R. *et al.* Array programming with NumPy. *Nature* **585**, 357–362 (2020).
62. Virtanen, P. *et al.* SciPy 1.0: Fundamental Algorithms for Scientific Computing in Python. *Nature Methods* **17**, 261–272 (2020).
63. pandas development team, T. pandas-dev/pandas: Pandas (2020). URL https://doi.org/10.5281/zenodo.3509134.
64. Hunter, J. D. Matplotlib: A 2d graphics environment. *Computing in Science & Engineering* **9**, 90–95 (2007).
